# Scalable Fabrication of Core–Sheath Nanofiber Yarns via NanoTwist Spinning for High-Performance Energy-Harvesting *E*‑Nanofiber Fabrics

**DOI:** 10.1021/acsami.5c04482

**Published:** 2025-06-17

**Authors:** Syamini Jayadevan, Akshaya Kumar Aliyana, George K. Stylios

**Affiliations:** Smart Wearable Electronics Group (SWEG), Research Institute for Flexible Materials, School of Textiles and Design, 3120 Heriot-Watt University, Galashiels TD1 3HF, U.K.

**Keywords:** nanoTwist spinning, nanofibers, core–sheath nanofiber yarns, nanofiber fabrics, electronic nanofiber fabrics, triboelectric nanogenerator

## Abstract

The fabrication of durable and scalable nanofiber fabrics (NFs) remains a critical challenge, limiting their practical applications in wearable electronics, smart textiles, biosensing, and energy harvesting systems. Recent advances in self-powered wearable textiles have demonstrated the potential of converting biomechanical motion into electricity, paving the way for battery-free next-generation SMART textiles. However, achieving a balance among flexibility, durability, high output performance, and wearability remains a major hurdle for real-world adoption. In this study, we introduce NanoTwist Spinning, an integrated nanospinning and yarn-twisting system designed to fabricate core–sheath nanofiber yarns (CSNYs) with high mechanical resilience and electrical conductivity. These yarns feature a precisely twisted nanofiber sheath wrapped around a conductive silver core, enabling large-scale processing through standard knitting machines to produce high-performance electronic-NFs (*E*-NFs). By optimizing fabrication parameters and utilizing polycaprolactone (PCL) and poly­(vinylidene fluoride-*co*-hexafluoropropylene) (PVDF-HFP) polymers, we achieved uniform, stable CSNYs with an optimized nanofiber wrapping rate of 38.21%. The resulting knitted NFs exhibited exceptional mechanical properties, including 83% compressive resilience, a breaking force of 350.5 N, a tensile strength of 17.53 MPa, and an elongation of 261.8%, ensuring superior durability, wearability, and comfort. To demonstrate real-world feasibility, the fabricated PCL/PVDF-HFP NF-based triboelectric nanogenerator (TENG) achieved an impressive electrical output of 100 V and 8 μA under real-time conditions, validating its potential for energy-harvesting applications. This work marks a significant breakthrough in scalable NYs and NFs production, offering a transformative pathway for the smart textile industry and opening new frontiers in sustainable, self-powered E-Textiles.

## Graphical abstract

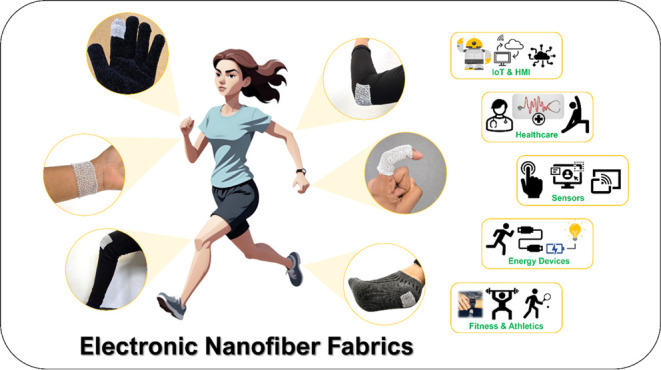

## 1 Introduction

The integration of nanotechnology and textile engineering has revolutionized the development of advanced nanotextiles, enabling innovations in smart fabrics, wearable electronics, and energy-harvesting devices.^1,2^ This progress has unlocked new possibilities for creating smart and functional textiles with enhanced performance. These advanced textiles are transforming sectors such as healthcare, sports apparel, automotive, and protective clothing by enabling innovative applications.^3,4^ For instance, in healthcare, nanotextiles are being used in drug delivery systems, wound dressings, tissue engineering, and smart fabrics capable of monitoring and sensing vital signals.^5^ In the automotive industry, nanotextiles are leading to lightweight, durable fabrics with enhanced safety features, pushing the boundaries of material performance and functionality. Common methods for producing nanotextiles include embedding nanoparticles into fibers, coating fabric surfaces, or employing electrospinning to create nanofiber-based yarns, typically in nonwoven mat structures.

Nanofiber-based textiles, including nanofiber mats, nanofiber yarns (NYs), and core–sheath (CS) yarns, offer superior mechanical strength, high surface area, enhanced porosity, and multifunctionality. The ability to engineer materials at the nanoscale with a high surface-to-volume ratio is the key to enhancing textile properties such as improved strength, durability, water repellence, stain resistance, wrinkle resistance, antibacterial activity, fire retardance, UV protection, conductivity, and sensing capabilities.^6–8^ These attributes have unlocked new frontiers in smart clothing, healthcare monitoring, electronic textiles, and sustainable energy generation.^9^ These advanced textiles are transforming sectors such as healthcare, sports apparel, automotive, and protective clothing by enabling innovative applications.^10–12^ Consequently, nanotextiles represent a paradigm shift in addressing challenges related to hygiene, comfort, performance, and sustainability in the textile industry.^13^ However, despite significant progress, scalability, durability, and mechanical stability remain key challenges, preventing the widespread adoption of nanofiber-based fabrics in real-world applications.

Ongoing effort focuses on developing nanofiber-based textiles, such as nanofiber mat, NYs, and CS yarns, exhibiting unique characteristics, such as superior mechanical strength, increased surface area, high porosity, and a variety of specialized functionalities.^14,15^ The goal is to precisely control their material properties and improve the performance for advanced functionalities. Recent advancements in nanofiber yarn fabrication have been achieved by introducing modifications to the conventional electrospinning setup, particularly in the collection process. Through the optimization of the collector geometries, different types of NYs, including twisted, aligned, and CS yarns, can be produced with enhanced performance. Among them, CSNYs have a broader range of applications compared to nanofiber yarns, revolutionizing multiple industries and enabling the next generation of intelligent, self-powered wearable technologies. CSNYs, composed of a central core encapsulated by a nanofiber sheath, integrate the properties of both components, thereby enhancing multifunctionality, but are regarded as NYs because their sheath is electrospun and twisted into a yarn.^16^ This hybrid structure represents a major leap forward in textile engineering, combining the strength and durability of traditional yarns with the advanced properties of nanofibers, such as superior electrical and electrochemical performance, antimicrobial capabilities, shape memory, sensing, triboelectric and piezoelectric functionalities, and making them ideal for diverse applications in healthcare, smart fabrics, electronic textiles, energy devices, and sensors.^17^ Joseph et al.^18^ and Wu et al.^19^ fabricated biodegradable electrospun CS micronanofibrous poly l-lactic acid (PLLA)/PCL yarns and polylactic acid (PLA)/poly­(lactic-*co*-glycolic acid) (PLGA) yarns, respectively, for biomedical tissue engineering applications. Recently, CSNYs featuring a conductive core and a nanofibrous sheath have gathered increasing attention in textile-based sensors and biomechanical energy harvesting devices, such as triboelectric and piezoelectric nanogenerators.^16^ Yang et al. developed a graphene-doped polyvinylidene fluoride (PVDF) nanofiber-wrapped carbon fiber CS yarn-based triboelectric textile, which was demonstrated to be effective for both energy harvesting and human movement monitoring.^20^ Stainless-steel conducting core yarns have also been utilized to fabricate PVDF,^21,22^ PLA,^23^ and polyamide 66 (PA66)^22^ nanofibrous CS yarns, respectively, for various energy harvesting and sensing applications. Similarly, Ma et al. made significant advancements by developing a micronano CS polyacrylonitrile (PAN)/PVDF composite nanofibrous yarn and woven nanofiber fabric (NF) for energy harvesting and sensing applications.^24^

Despite significant progress in the fabrication of CSNYs, the lack of a scalable fabrication process for producing uniform, durable CSNYs has hindered their commercialization.^14^ Consequently, researchers continue to develop their own custom-designed and modified electrospinning techniques to fabricate CSNYs for energy harvesting applications. In many cases, commercially available yarns produced through various techniques, each offering distinct characteristics such as diameter, conductivity, flexibility, and mechanical properties, are employed as the core material. Given that the core predominantly governs the electrical performance and mechanical integrity of the final CS structure, a comprehensive investigation of the mechanical behavior of these yarns and fabrics is crucial before their integration into wearable garments. Since energy-harvesting textiles are subjected to repeated mechanical stresses during human motion, critical properties such as flexibility, comfort, mechanical resilience, and tensile strength must be thoroughly assessed. Additionally, the influence of different fabric structures and knitting or weaving patterns on the mechanical performance of the overall fabric is a vital factor to consider, as structural design can significantly affect the fabric’s ability to endure cyclic deformation without mechanical degradation.

This account discusses the fabrication challenges, the role of core material selection, the impact of fabric structural design, and the essential mechanical evaluations necessary for the development of durable and wearable energy harvesting systems. To address these challenges, we introduce NanoTwist Spinning, an integrated electrospinning and yarn-twisting system designed to fabricate continuous, high-performance CSNYs. This approach enables precise control over nanofiber deposition and twisting, ensuring the formation of uniform CS structures with enhanced mechanical stability, electrical conductivity, and processability. Using polycaprolactone (PCL) and poly­(vinylidene fluoride-*co*-hexafluoropropylene) (PVDF-HFP), we successfully developed CSNYs with an optimized nanofiber wrapping rate of 38.21%, demonstrating their ability to be processed into knitted NFs using standard textile manufacturing techniques. This study presents a significant advancement in scalable NY and NF production with a strong emphasis on durability, wearability, and real-world integration. By leveraging the triboelectric properties of PCL and PVDF-HFP CSNYs, we demonstrate the fabrication of a wearable NF-TENG, capable of achieving 100 V and 8 μA under real-time conditions of 8 N and 4 Hz. The findings pave the way for the development of self-powered smart textiles, opening new possibilities in energy-harvesting electronic fabrics, biomedical monitoring, and next-generation wearable technology.

## 2 Materials and Methods

### 2.1 Materials

Polycaprolactone (PCL; average Mn ∼80,000) and poly­(vinylidene fluoride-*co*-hexafluoropropylene) (PVDF-HFP; average Mn ∼110,000) were purchased from Sigma-Aldrich. Organic solvents such as dichloromethane (DCM), *N*,*N*-dimethylformamide (DMF), and acetone were bought from Fisher Scientific, UK, and Rathburn Chemicals Ltd., Scotland, respectively. All of these chemicals were used without any further purification. Conducting core yarn (*C*-yarn) made of silver-coated nylon microfibers (117/17 × 2 HCB) was procured commercially.

### 2.2 Fabrication of CSNYs via the NanoTwist Spinning System

The detailed fabrication process of the CSNYs involves twisting or wrapping a core fiber with an aligned sheath of nanofibers, typically achieved through the NanoTwist Spinning system.^14^ In this work, we developed a NanoTwist Spinning system designed to produce continuous CSNY, as depicted in Figure 1. The system includes key components such as a rotating funnel, yarn holder, and collector integrated with an existing electrospinning unit, comprising a high-voltage source, syringe pump, and a spinneret. The funnel rotator, constructed from highly conductive copper with an outer diameter of 90 mm and an inner diameter of 70 mm, ensures uniform twisted fiber deposition onto the *C*-yarn. Synchronizing the yarn take-up speed with the funnel rotator is critical for maintaining consistent tension and uniform nanofiber wrapping and twisting. A critical distance is maintained between the spinneret tip and the funnel collector, with an angle of 40° to the central axis of the funnel and the *C*-yarn for maximum nanofiber deposition, wrapping, and consistent twisting, which are crucial for the durability of the produced NYs.

**Figure 1:**
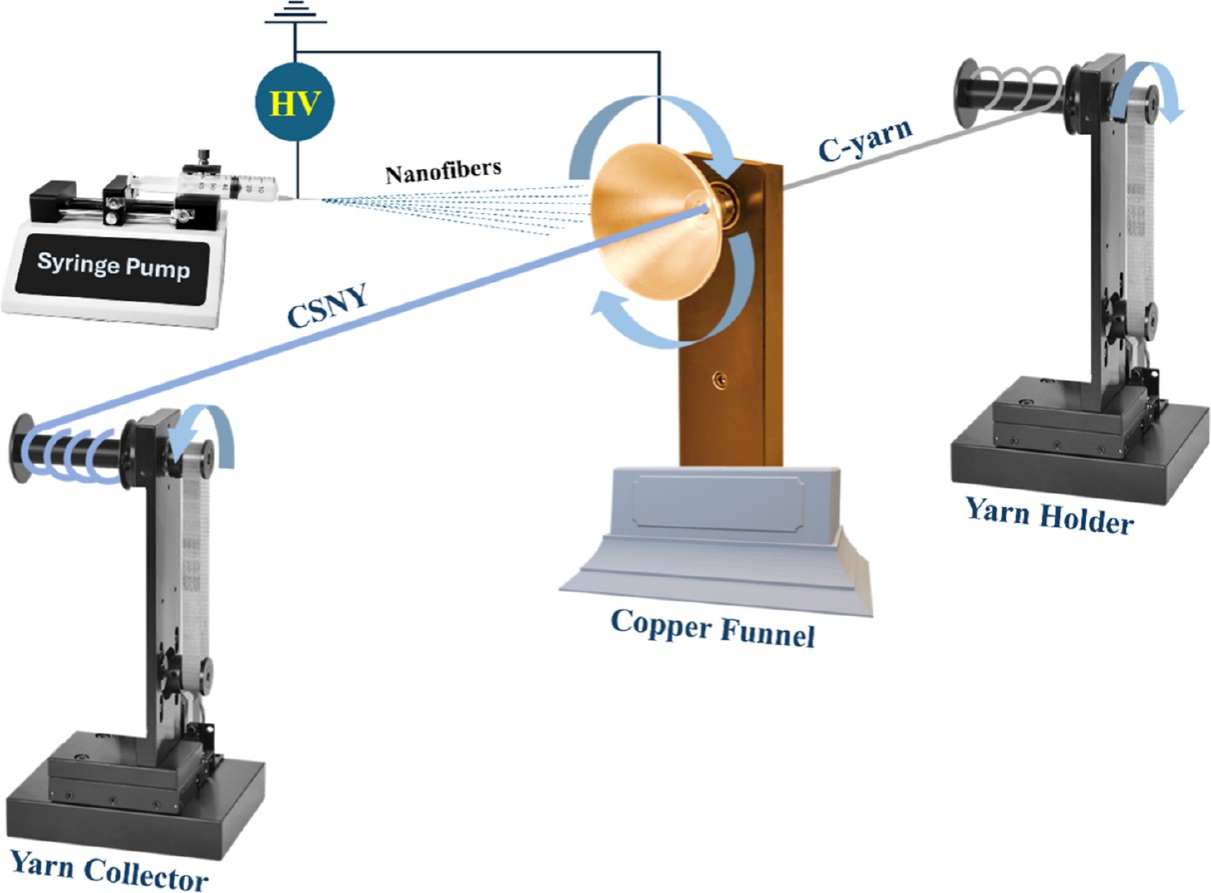
Schematic illustration of the NanoTwist Spinning, an integrated electrospinning and yarn-twisting and collecting system designed to fabricate continuous CSNYs.

### 2.3 Optimization of Nanospinning and Yarn-Twisting Parameters

Fifteen wt % PCL was dissolved in a DCM/DMF mixture, with a molar ratio of 1:1, via magnetic stirring for 10 h under room temperature (Figure 2a (i)). Similarly, 15 wt % PVDF-HFP was dissolved in a 1:1 solvent mixture of DMF/acetone, by 24 h of magnetic stirring at room temperature (Figure 2a (ii)). The prepared polymer solution was loaded into a 10 mL plastic syringe with an outer diameter of 15 mm and fitted with an 18G stainless-steel spinneret having an outer diameter of 1.2 mm. Positive and ground electrodes were connected to the spinneret and the rotating copper funnel, respectively. The yarn holder and yarn collector were positioned 80 and 40 cm away from the funnel collector, respectively. The CSNY fabrication process is schematically represented in Figure 2b. The flow rate was adjusted between 0.5 and 1.5 mL/h, and the needle distance was varied from 10 to 12 cm to achieve a continuous nanofiber cone (Figure 2c) and uniform nanofiber wrapping around the *C*-yarn. The fabricated PCL CSNY was left undisturbed at room temperature overnight to allow for residual solvent evaporation, and then it was transferred to a plastic yarn cone (Figure 2d,e). For PVDF-HFP CSNY, the drying process involved keeping the yarn in an oven set to 60 °C overnight (Figure 2f).

**Figure 2:**
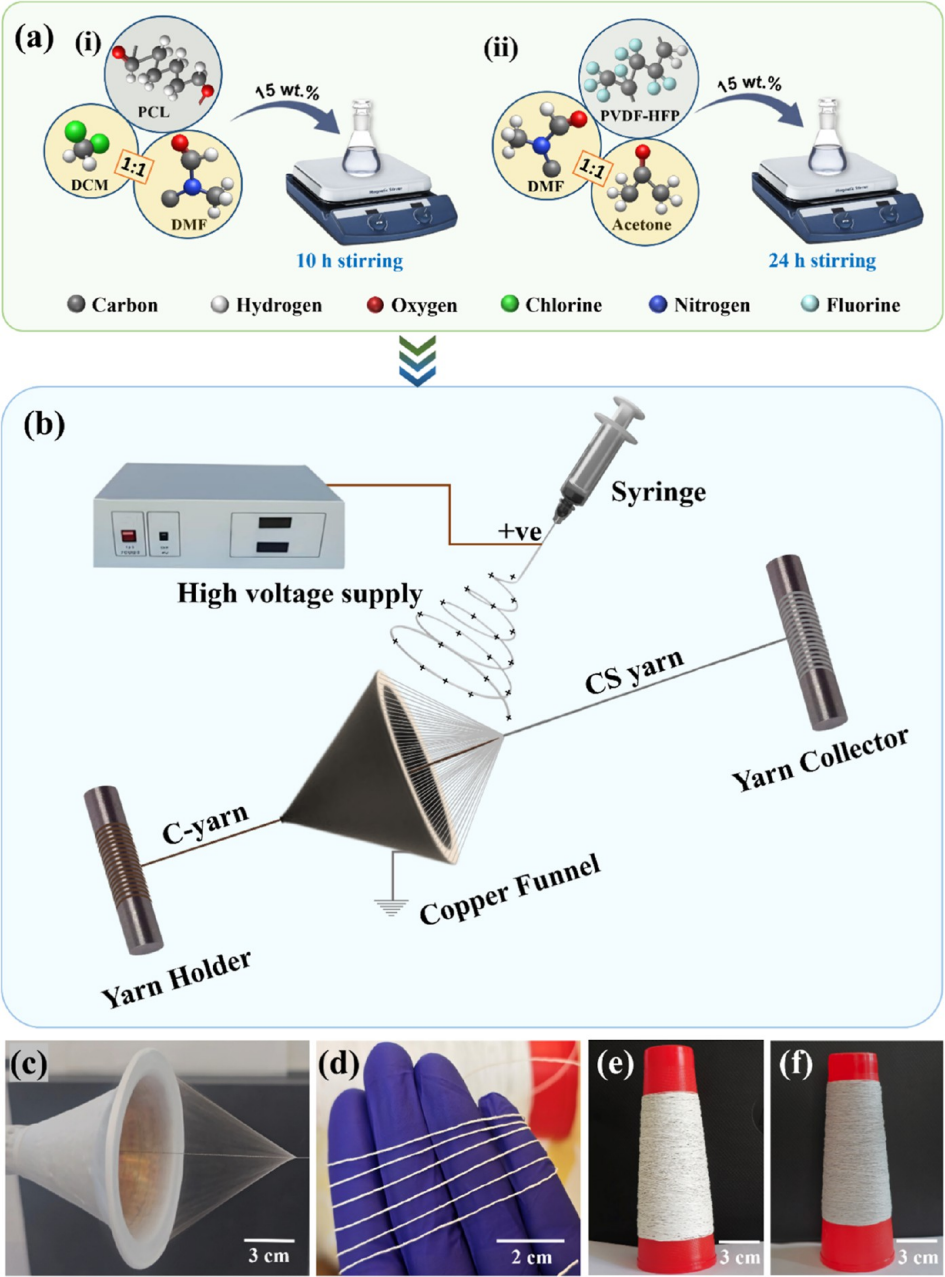
Schematic representation detailing the steps involved in the fabrication of NYs: (a) Preparation of polymer solutions, such as (i) PCL and (ii) PVDF-HFP, via magnetic stirring, and (b) nanofiber spinning, yarn-twisting, and collecting process via the NanoTwist Spinning system. (c) Photograph of the nanofibrous cone wrapping nanofibers around the *C*-yarn during electrospinning. (d) PCL CSNY. The spool of (e) PCL and (f) PVDF-HFP CSNY.

### 2.4 Development of Knittable NFs

NFs were fabricated using a Dubied V bed 10 gg knitting machine (i.e., 10 needles per inch) with a yarn tension of 10. The NY was supplied to the knitting machine using a cone-shaped bobbin, collected by the mechanism shown in Figure 1, which facilitates smooth unwinding during the knitting process. 1 × 1 rib, 2 × 2 rib, single-bed (SB), double-bed (DB), half cardigan (HC), and full cardigan (FC) NFs were made using the same knitting machine. The 1 × 1 and 2 × 2 rib patterns feature alternating knit and purl stitches, resulting in a stretchy, textured fabric with vertical columns of knit stitches alternating with columns of purl stitches. SB knitting utilizes one set of needles to produce fabrics with a flat and uniform appearance, while DB knitting employs two sets of needles, enabling the creation of more complex and densely structured fabrics with interloping patterns. The HC pattern combines knitting on one side and purling on the other, incorporating additional stitches or rows to create a textured, bulky fabric. The FC stitch is a more intricate rib-like pattern, characterized by an interlacing of knit and purl stitches that produces a highly textured and thick fabric.

### 2.5 Development of NF-TENG

The obtained PCL and PVDF-HFP knitted NF (total area: 27 cm^2^) with excellent mechanical resilience and flexibility was used as two triboelectric materials for mechanical energy harvesting under the contact separation mechanism. PCL and PVDF-HFP nanofibrous sheaths of the NFs were regarded as tribopositive and tribonegative materials, respectively. The silver-coated nylon microfibrous *C*-yarn acts as the inner electrode of the NF-TENG. The ends of the electrode (*C*-yarn) from PCL and PVDF-HFP NFs were connected to the external circuit for measurements. The effective area of NF coming into contact during the pressing and releasing cycle is 4 cm^2^.

### 2.6 Structural and Mechanical Characterization

The surface morphologies of the *C*-yarn and CSNY were analyzed using a Hitachi S-4300 SEM and Quanta 650 FEG scanning electron microscope (SEM) with an accelerating voltage of 10 kV. ImageJ software was used to measure the diameter distribution of nanofibers and yarns, which was calculated by measuring a minimum of 100 nanofibers. The surface profile of the yarns and knitted fabrics was analyzed by using an Alicona Infinite Focus 5G 3D microscope. A precision LCR meter (LCR-6002) was used to study the electrical properties of the yarns and fabrics. Fourier transform infrared (FTIR) analysis of *C*-yarn and CSNYs was performed by using a Nicolet iS5 ATR-FTIR spectrometer. A universal tensile testing machine (Instron 2519) was used to measure the mechanical properties, such as tensile strength and maximum breaking force, of *C*-yarns and CSNYs. First, the yarns were cut into 25 cm long segments, and both ends of the samples were mounted onto the metallic grip holder clamp at a gauge length of 5 cm using a load cell of 5 kN. The sample was tested at a crosshead speed of 100 mm/min under a strain rate of 0.2% per second. All mechanical property tests of the yarns were conducted at a standard atmosphere for textile testing in a conditioned room at 20 °C ± 2 °C (68 °F ± 4 °F) temperature and 65% ± 4% relative humidity. The tests were repeated five times on separate samples before taking an average. The compression recovery performance of the knitted NF structures was measured by an Instron Test Machine 2519 system with a 5 kN load cell at a compression rate of 3 mm/min to ascertain their wearability and comfort. The diameters of the top and bottom circular metal plates were 4.5 cm. The tensile properties of the SB and DB knitted in the course and wale direction were studied using the Instron tensile testing machine. Both ends of the fabric samples (25 × 50 mm^2^) were mounted onto the metallic holder at a gauge length of 3 mm and tested at a crosshead speed of 60 mm/min. The extensibility of both NFs in the wale and course directions at various loads (4.9, 19.6, and 98.1 N/m) were measured using an Extension Meter of the Fabric Assurance by Simple Testing (FAST) instrument. All fabric mechanical property tests were conducted at a standard atmosphere for textile testing in a conditioned room at 20 °C ± 2 °C (68 °F ± 4 °F) temperature and 65% ± 4% relative humidity. Contact angle measurements were performed on an Attention Optical Tensiometer Theta Lite contact angle meter at room temperature. An aqueous detergent solution was prepared by mixing 4 mL of detergent with 800 mL of lukewarm water. Both SB (15 × 15 cm^2^) and DB (11.5 × 7 cm^2^) NFs were washed separately for 10 min using a magnetic stirrer at a rotation speed of 300 rpm. Then, the fabric was rinsed by hand in tap water to remove any detergent remains and then air-dried at room temperature. The electrical output performance and the capacitor charging measurements were performed by using a low-noise current preamplifier (SR570, Stanford Research) and a digital storage oscilloscope.

## 3 Results and Discussion

### 3.1 Fabrication and Structural Characterization of CSNYs

We designed and developed a combined nanospinning, yarn-twisting system for the fabrication of CSNYs (Video S1). To evaluate the performance of the system, we initially started with electrospinning PCL, known for its biocompatibility and biodegradability, which are essential features for wearable nanotextile applications. The resultant PCL CSNY consists of a conducting microfibrous *C*-yarn encapsulated with a continuous nanofibrous PCL twisted sheath of nanofibers. The silver-coated nylon two-ply microfiber yarn, shown in Figure 3a, was used as a core component.

**Figure 3:**
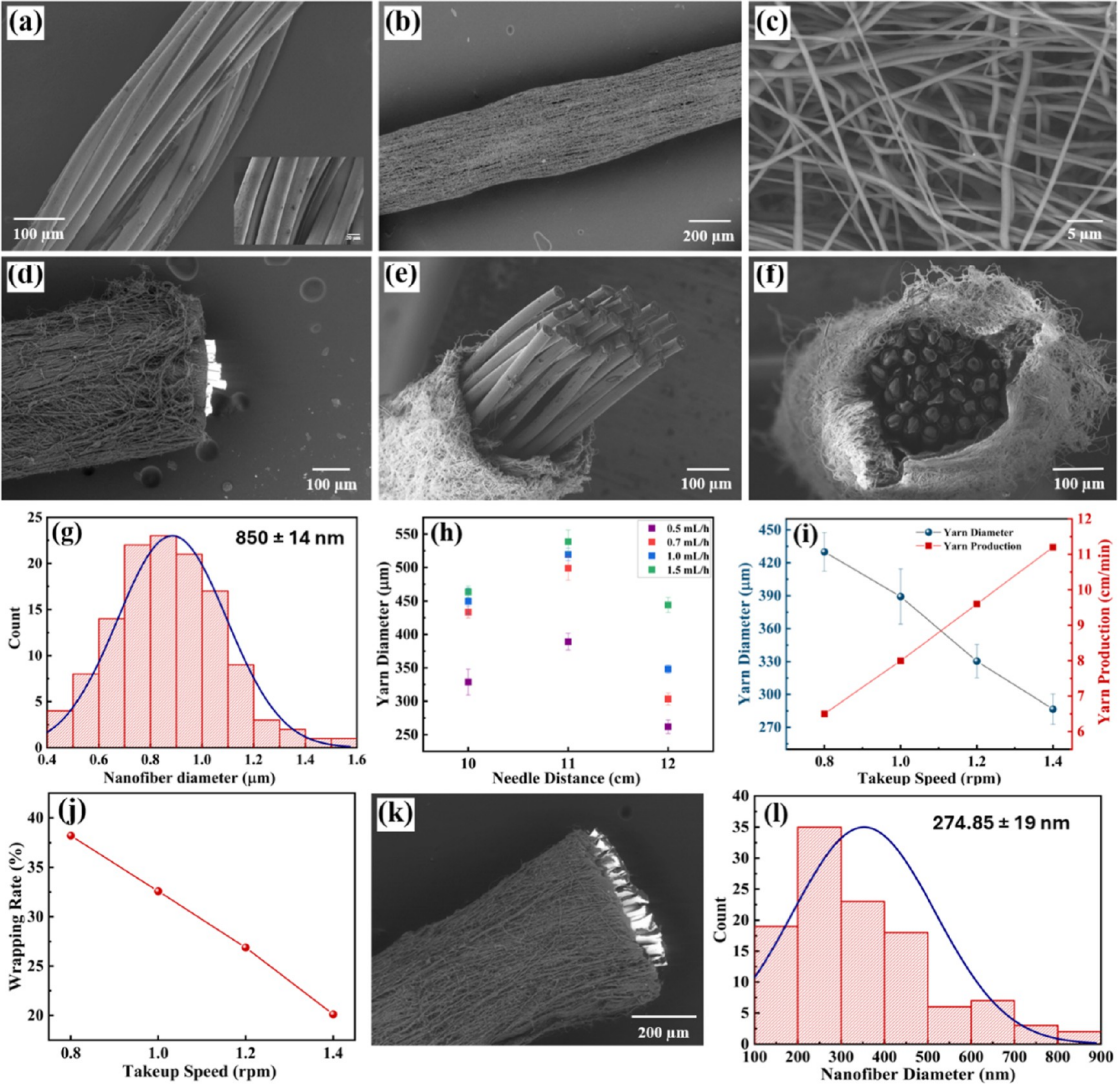
Scanning electron microscopic (SEM) images of (a) *C*-yarn (inset shows the SEM image at higher magnification), (b) PCL CSNY, (c) Nanofibrous surface of PCL CSNY, and (d–f) cross-sectional SEM of the PCL CSNY at different orientations. (g) Statistical diameter distribution of PCL nanofibers. (h) Average PCL CSNY diameter at different needle distances and flow rates. Effect of take-up speed on the PCL CSNY’s (i) diameter and yarn production rate and (j) nanofiber wrapping rate. (k) Cross-sectional SEM image of PVDF-HFP CSNY. (l) Statistical diameter distribution of PVDF-HFP nanofibers.

The fabrication of continuous and uniform CSNY is governed by key electrospinning parameters, including applied voltage, flow rate, needle-to-collector distance, funnel rotation speed, and take-up speed. These parameters must be optimized for each polymer system. The high-voltage power supply induces current flow through the polymer solution via a charged metallic spinneret, generating spherical droplets that deform to cone-jet mode. At a critical voltage, these droplets elongate to produce ultrafine nanofibers. This critical value of applied voltage varies from polymer to polymer. Here, we conducted electrospinning under a voltage range of 7–14 kV. At a higher voltage of 14 kV, the nanofiber wrapping became nonuniform and sparse, while 9 kV was identified as the optimal voltage for producing a stable nanofiber cone that yielded thick and uniform wrapping around the conductive *C*-yarn (Figure S2). Excessive high voltages hindered proper deposition of nanofibers onto the grounded copper funnel collector, resulting in inconsistencies. Furthermore, the funnel rotation speed and take-up speed were optimized to ensure uniform nanofiber coverage, with an effective funnel rotation of 85 rpm leading to tightly wrapped CSNY. The distance from the needle to the funnel collector, along with the flow rate of the polymer solution through the spinneret, also significantly influenced the morphology and wrapping of the nanofibers. To assess these effects, the PCL solution was electrospun from three different needle positions across four flow rates, with the spinneret angled at 40° from the central axis of the rotating funnel collector. The PCL nanofiber diameter and morphology of the NYs were determined using SEM analysis, and the average nanofiber diameter observed by varying the needle distance and solution flow rate is shown in Table 1 (Figures S3 and S4). The SEM images of PCL CSNYs produced under different needle distances and flow rates are shown in Figure S5. From Table 1, it is evident that the nanofiber diameter was easily affected by the needle distance and flow rate. PCL CSNYs with uniform and finer nanofiber wrapping were formed when the needle distance was 11 cm and the flow rate 0.7 mL/h (Figure 3b–f. Large-diameter nanofibers were formed when the needle distance was kept close to the funnel collector. When the needle was 10 cm away from the funnel, fibers with a diameter of around 3000 nm were more likely to be produced. In contrast, the diameter of the nanofiber decreased as the distance was increased, depending on deposition time and evaporation rate. When the distance was increased to 11 cm and then 12 cm, a considerable decrease in the nanofiber diameter was observed. Similarly, a critical solution flow rate was required to maintain a balance between the leaving solution and the replacement of that solution with a new one during jet formation at the tip of the spinneret, ensuring a stable jet cone formation.^25^ An increase or decrease in the flow rate may lead to an unstable jet, forming nanofibers with a wide range of diameters. Furthermore, an increase in the diameter of the nanofibers with an increase in the flow rate can also be attributed to the nonevaporation of the solvent and low stretching of the solution during flight between the needle and collector.^26^ The PCL solution electrospun under these optimum conditions resulted in the formation of uniform and defect-free nanofibers with an average PCL nanofiber diameter of 850 ± 14 nm and CSNY diameters of 498.82 ± 17.6 μm (Figure 3g,h). The diameter of the CSNYs can be easily adjusted by varying the take-up speed, which will also affect the production rate of the yarn, as shown in Figure 3i. The increase in the take-up speed results in a reduced diameter of the CSNY due to less nanofiber wrapping (Figure S6). The wrapping rate (*R*_w,_ in %) of the CSNY can be calculated using eq 1.^27^

1 $$R_{w}=\frac{\left(M_{cs}-M_{c}\right)}{M_{c}}\times 100$$

where *M*_cs_ and *M*_c_ are the mass of 1 m long CSNY and *C*-yarn, respectively.

**Table 1:** Effect of Nanofiber Diameter on Needle Distance and Solution Flow Rate

		needle position		
		10 cm	11 cm	12 cm
flow rate	0.5 mL/h	1127.51 ± 55 nm	944.21 ± 58 nm	1021.72 ± 45 nm
	0.7 mL/h	1065.82 ± 69 nm	850 ± 14 nm	817.27 ± 37 nm
	1 mL/h	795.45 ± 296 nm	1068.36 ± 42 nm	990.69 ± 75 nm
	1.5 mL/h	592.66 ± 152 nm	1204.73 ± 87 nm	1009.78 ± 43 nm

The yarn was collected at a minimum take-up speed of 0.8 rpm, to attain a maximum nanofiber deposition with an average wrapping rate of 38.21% and a production rate of 6.5 cm/min, as shown in Figure 3i,j. By increasing the take-up speed to 1.4 rpm, the production rate of the CSNY can be increased to 11.2 cm/min with a reduced wrapping rate of 20.1%.

To evaluate the material compatibility and versatility of the NanoTwist Spinning system, it was important to demonstrate the successful fabrication of CSNYs using different types of polymer materials with varying chemical and physical properties. Since the intended application is in TENGs and PCL is triboelectrically positive, the second material was selected to complement this behavior based on electron affinity. PVDF-HFP, known for its strong electron-withdrawing capability, was chosen to create an opposite triboelectric polarity, which enhances charge separation during contact and separation cycles, thereby improving the energy harvesting performance. The electrospinning conditions and solution parameters for PVDF-HFP were maintained the same as those used for PCL, except for the flow rate, which was reduced to 0.5 mL/h due to the lower viscosity of the 15 wt % PVDF-HFP solution compared to PCL. Figure 3k shows the cross-sectional SEM image of PVDF-HFP CSNY with an average yarn diameter of 467.5 ± 41 μm and an average PVDF-HFP nanofiber diameter of 274.85 ± 19 nm (Figure 3l). This demonstrates the efficiency and adaptability of the system in processing different polymers, confirming its potential in fabricating diverse nanofibrous CS yarns for NF manufacturing.

### 3.2 Mechanical Properties and Durability of CSNYs

The exceptional textile-like properties of the CSNYs are demonstrated by their ability to bend, braid, and knot. The excellent bending and flexing ability of the yarn was illustrated by making a simple floral pattern, as shown in Figure 4a. The robustness of the yarn allows it to form a two-plied and braided structure, as shown in Figure 4b,c, respectively, without compromising its structural integrity. Furthermore, the ability of the yarns to be effectively knotted is displayed in Figure 4d,e. The combination of flexibility, strength, and knotting makes the yarn suitable for producing various embroidery patterns. A chain stitch embroidery design “SWEG” and various embroidery stitches created using PCL CSNY are shown in Figures 4f and S7, respectively, highlighting its potential for use in conventional, decorative, and functional textile applications. The resistance variation of a 10 cm long yarn under different mechanical deformations, such as straight, bent, and knotted, is shown in Figure 4g. The yarn shows a slight increase in its resistance from 18.36 to 21.4 Ω when subjected to bending and knotting, indicating robust conductive properties, highlighting its potential in applications such as flexible electronics and wearable devices, where reliable electrical performance is crucial to withstand repeated mechanical deformations without substantial loss of conductivity.

**Figure 4:**
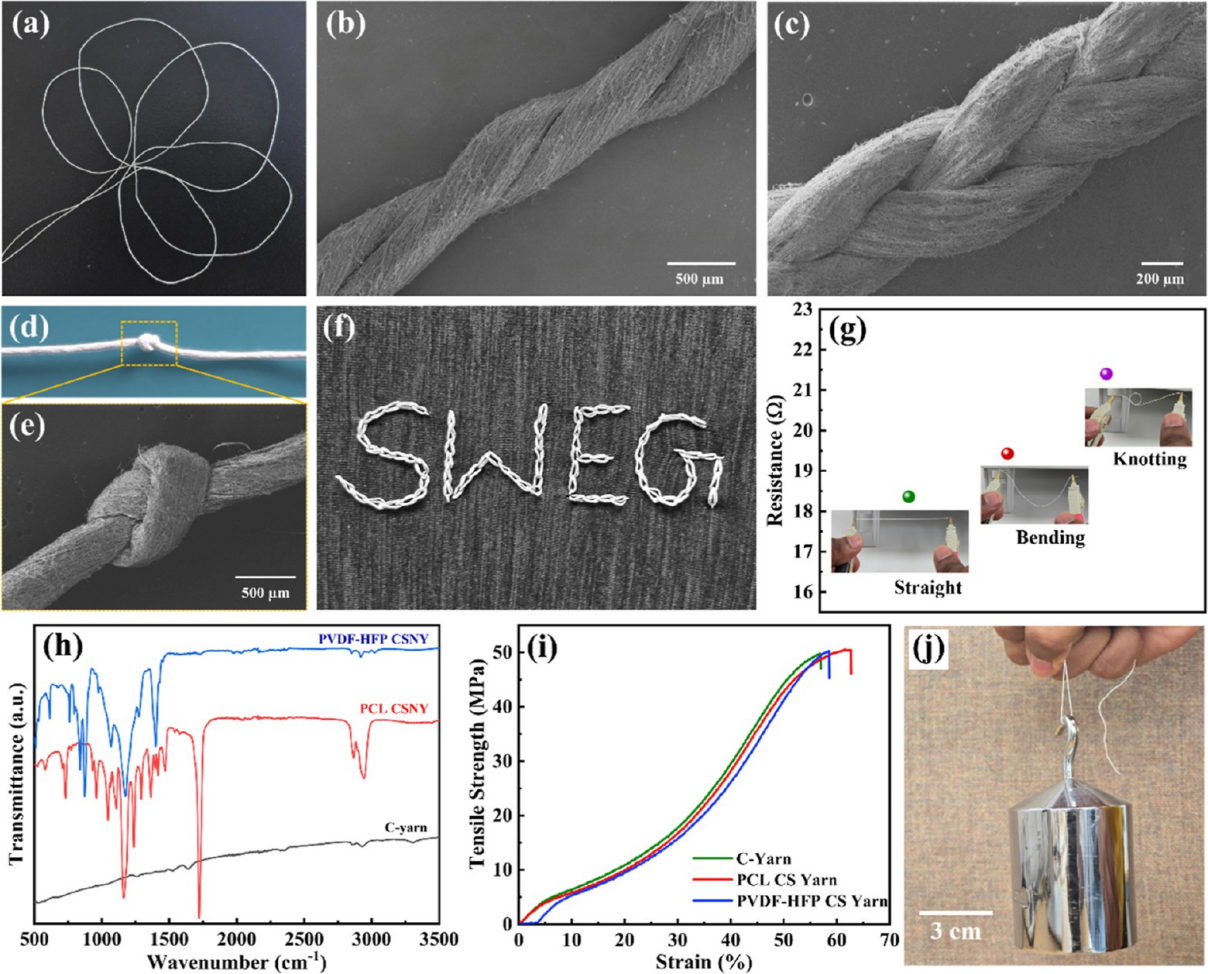
(a) Illustration of the flexibility of CSNY by forming a simple floral pattern. SEM image of a PCL CSNY-based (b), two-plied, and (c) braided structure. (d) Photograph and (e) SEM image of the knotted yarn. (f) Photograph of chain stitch embroidery pattern “SWEG” produced using PCL CSNY. (g) The electrical resistance of the PCL CSNY under mechanical deformations. (h) FTIR spectra of *C*-yarn and PCL and PVDF-HFP CSNYs. (i) Tensile properties of *C*-yarn and CSNYs. (j) Photograph of PCL CSNY holding a weight of 1 kg without breaking.

FTIR spectra of *C*-yarn and CSNYs in the range of 500–3500 cm^–1^ were analyzed, as shown in Figure 4h. The FTIR spectrum of *C*-yarn exhibits characteristic peaks at 3306 cm^–1^, 2932 cm^–1^, 1643 cm^–1^, and 1524 cm^–1^, which correspond to amine stretching, CH_2_ stretching, amide carbonyl stretching, and N–H bending vibrations, respectively.^28^ The prominent characteristic peaks at 1722 cm^–1^ and 1163 cm^–1^ in the FTIR spectrum of PCL CSNY are associated with the stretching vibrations of carbonyl (C=O) and C–O–C groups, respectively, indicating the presence of strong electron-donating functional groups.^29^ In the FTIR spectrum of PVDF-HFP CSNY, the three distinct peaks at 1397 cm^–1^, 1172 cm^–1^, and 839 cm^–1^ correspond to –CH_2_ bending vibrations, CF_2_ stretching, and CF_2_ rocking, respectively, confirming its strong electron-withdrawing nature.^30^ The complementary electronic properties of PCL CSNY as a strong electron donor and the electron-withdrawing nature of PVDF-HFP CSNY make them a perfect tribopositive–tribonegative pair for efficient energy generation in triboelectric applications.

The fineness and mechanical strength of textile fibers or yarns are crucial indicators of their quality. To underscore the potential of these NYs in traditional textile manufacturing, we investigated the basic mechanical properties of the *C*-yarn and CSNY. As expected, CSNY demonstrated properties nearly identical to those of the *C*-yarns. The *C*-yarn has a linear density of 31.4 tex, while PVDF-HFP and PCL CSNY have linear densities of 35.2 and 39.7 tex, respectively, demonstrating that the nanofibrous PCL sheath is extremely lightweight, with the core contributing much of the weight of the whole yarn. The tensile measurements revealed that the tensile strength and strain at break of the CSNY were slightly higher than those of the *C*-yarn, yet similar (Figure 4i), as expected. The PCL CSNY achieved a tensile strength of 50.5 MPa and an elongation of 62.7%. As shown in Figure 4h, the CSNY can hold a weight of 1 kg, showcasing its remarkable robustness and load-bearing capacity. These results confirm that the CSNY not only meets but potentially exceeds the performance of commercial yarns, affirming its suitability for further textile fabrication.

### 3.3 Knitted NF Formation and Textile Performance

Although there are numerous reports on the fabrication and application of CSNY and CSNY-based nanotextiles in various domains, such as smart-electronic high-performance nanotextiles, textile-based energy devices, sensors, and medical devices, there is a notable lack of studies addressing the durability, practicality, wearability, and mechanics of these fabrics. Most of these attempts have not used industrial machines to make industry-standard knitted or woven fabrics. Since nanotextile-based devices often interact with complex and irregular human body stimuli, user comfort, wearability, and good body fitting are critical. Smart nanotextiles, which are worn close to the skin, require fabrics with good properties to ensure a better fit [33]. A precise understanding of fabric mechanical properties—such as extension and compression—is essential to ensure that materials meet overall comfort, performance, and quality standards. This is particularly important for nanotextile-based sensors and energy devices, which depend on the biomechanics of the human body for their proper functionality. Mechanical properties like compression resilience, extensibility, and bendability impact how the fabric performs under various conditions and stresses with repeated use, which is crucial for ensuring that smart textiles maintain their functionality over their intended lifespan. And finally, washability is another key factor, as smart nanotextiles must withstand regular cleaning without degrading their stability and performance. This multidisciplinary approach is crucial for the successful integration of advanced textile technologies into real-world applications. Thus, a comprehensive understanding of these properties is essential for advancing the practical application and performance of the nanotextiles.

Knitting is a fundamental technique in the textile industry, renowned for its unique characteristics such as natural stretch and flexibility, which contribute to a perfect fit and exceptional comfort. All fabricated CSNYs were tested and confirmed to be suitable for knitting into various structures, including 1 × 1 rib, 2 × 2 rib, SB, DB, HC, and FC, as illustrated in Figure 5. The knitting process involves stresses and strains from loop formation and takedown tension, which impose additional demands on yarn strength, flexibility, and durability. Notably, no special precautions were taken during the knitting of these yarns despite the aggressive stretching and looping that occurs during loop formation (see Video S2).

**Figure 5:**
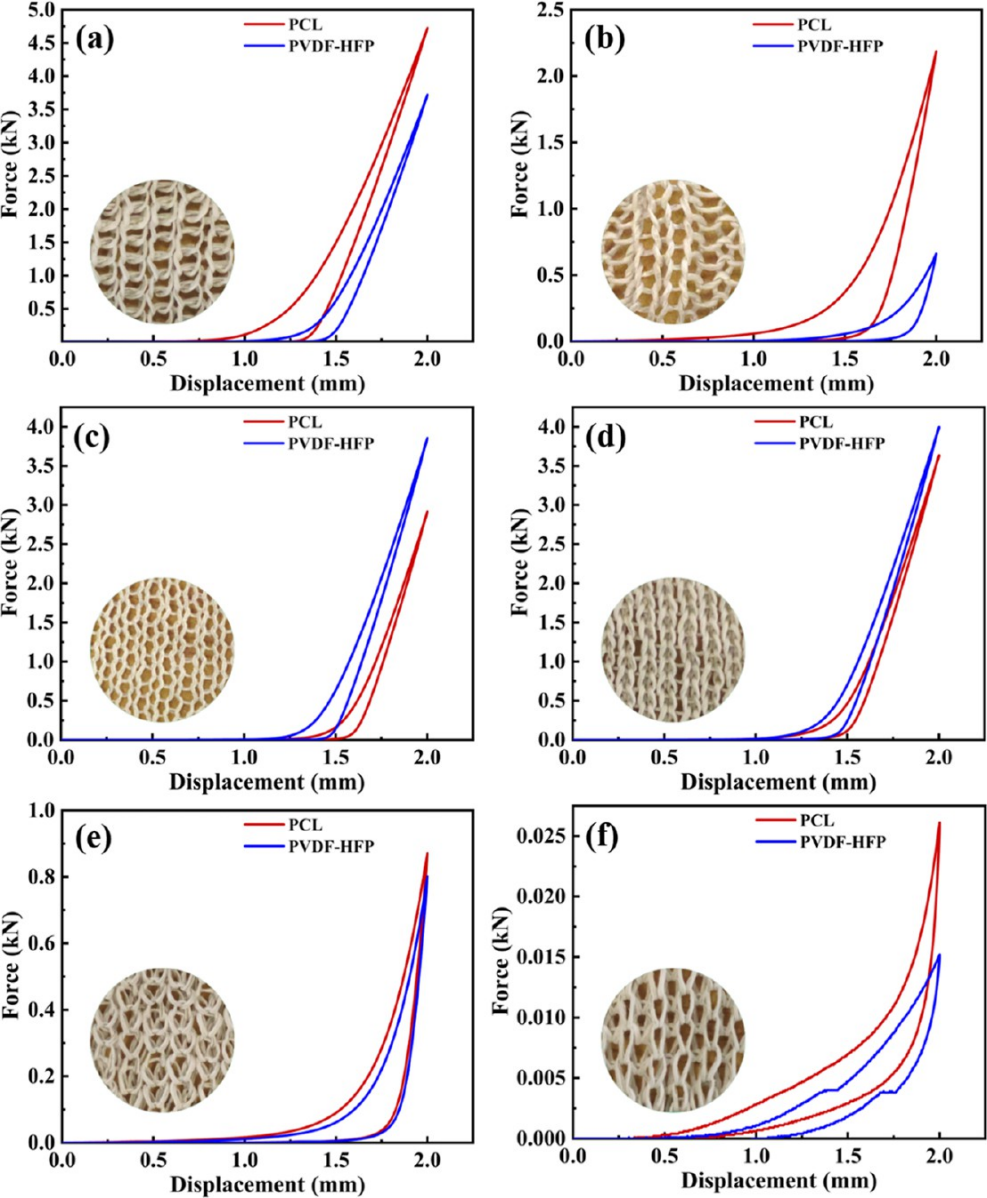
Fabric compression recovery curve of PCL- and PVDF-HFP-based knitted NF structures: (a) 1 × 1 rib, (b) 2 × 2 rib, (c) SB, (d) DB, (e) HC, and (f) FC. The inset shows the microphotographs of various knitted structures.

Fabric compressibility is a critical aspect of material characterization, particularly in assessing how fabrics respond to compressive forces. This testing is vital for evaluating the structural integrity, comfort, and performance of materials, ensuring their suitability for specific end uses.^31^ Compression tests were initially conducted on both PCL and PVDF-HFP CSNY-based knitted structures to determine their fabric-like behavior. Figure 5a–f shows the compression recovery curve of PCL and PVDF-HFP-based knitted NF structures. Their compression recovery performance was evaluated by calculating compressive energy (*W*_C_), compressive resilience (*R*_C_), and compressive linearity (*L*_C_), which are shown in Figure 6a–c, respectively. The compressive energy is the total work done or energy absorbed by the fabric during compression, reflecting the resistance offered by the fabric to deformation under compressive loads, and can be determined by eq 2.^32^

2 $$W_{C}=\int_{0}^{D_{\max}}Fdx$$

where *D*_max_ is the maximum displacement, and *F* denotes the force during compression (Figure S8b). A higher compressive energy value means that the fabric can absorb more energy before it deforms, while a lower value means less energy. Fabrics with high compressive energy are generally more resistant to compression and can endure higher forces before they compress, indicating that the fabric is likely to be more durable and retain its shape/structure over time. Fabrics with low compressive energy tend to deform more easily under load and are suitable for applications requiring more flexibility and softness, where the fabric is not expected to bear heavy loads or resist significant compression.^33^ PCL-based 1 × 1 rib NF has the highest compression energy of 1.84 J, and the FC NF made with PCL (0.009 J) and PVDF-HFP (0.006 J) shows the lowest compression energy. Compressive linearity describes the relationship between the applied compressive force and the resulting deformation of the fabric and can be obtained from eq 3.^34^

3 $$L_{C}=\frac{W_{c}}{\frac{1}{2}F_{\max}D_{\max}}$$

where *F*_max_ denotes the maximum compressive force. It measures the elasticity of fabrics, with a value of 1 indicating perfect elasticity. However, since fabrics are not fully elastic, their *L*_C_ values generally lie between 0 and 1.^35^ PCL and PVDF-HFP-based NFs show a compressive linearity of less than 0.41, indicating that they are typical fabrics. Among these, PVDF-HFP FC NF presents the highest *L*_C_ value of 0.405. Compressive resilience measures the ability of a fabric to recover its original shape after compression. A higher compressive resilience means that the fabric can absorb energy during compression and release most of it upon decompression, making it ideal for applications where shape retention and longevity are important. Fabrics with higher compressive resilience are generally more comfortable because they not only conform to the body but also provide a soft and supportive feel. Compressive resilience can be calculated using eq 4 and is expressed as a percentage.^35^

4 $$R_{C}=\frac{\int_{o}^{D_{\max}}F^{\prime }dx}{W_{C}}\times 100$$

where *F* ^′^ denotes the force during decompression. SB and DB NFs made from both PCL and PVDF-HFP CSNY exhibited excellent compressive resilience, above 82%. It means that after decompression, they can recover 82% of their original structure. Since the SB and DB exhibited excellent compression resilience, larger knitted NF samples were created with SB and DB structures using PCL CSNY. The effective area of SB was 16 × 15 cm^2^, while for DB, it was 11.5 × 7 cm^2^ (Figure S9). Figure 6d,e display the PCL-based SB and DB NFs, respectively. The high-density PVDF-HFP-based DB NF wrapped around the finger, exhibiting excellent flexibility, softness, and comfort on the skin, is shown in Figure 6f,g. The photograph of the NF formed around a yarn-winding cone and covering a hand finger is shown in Figure 6h,i, respectively.

**Figure 6:**
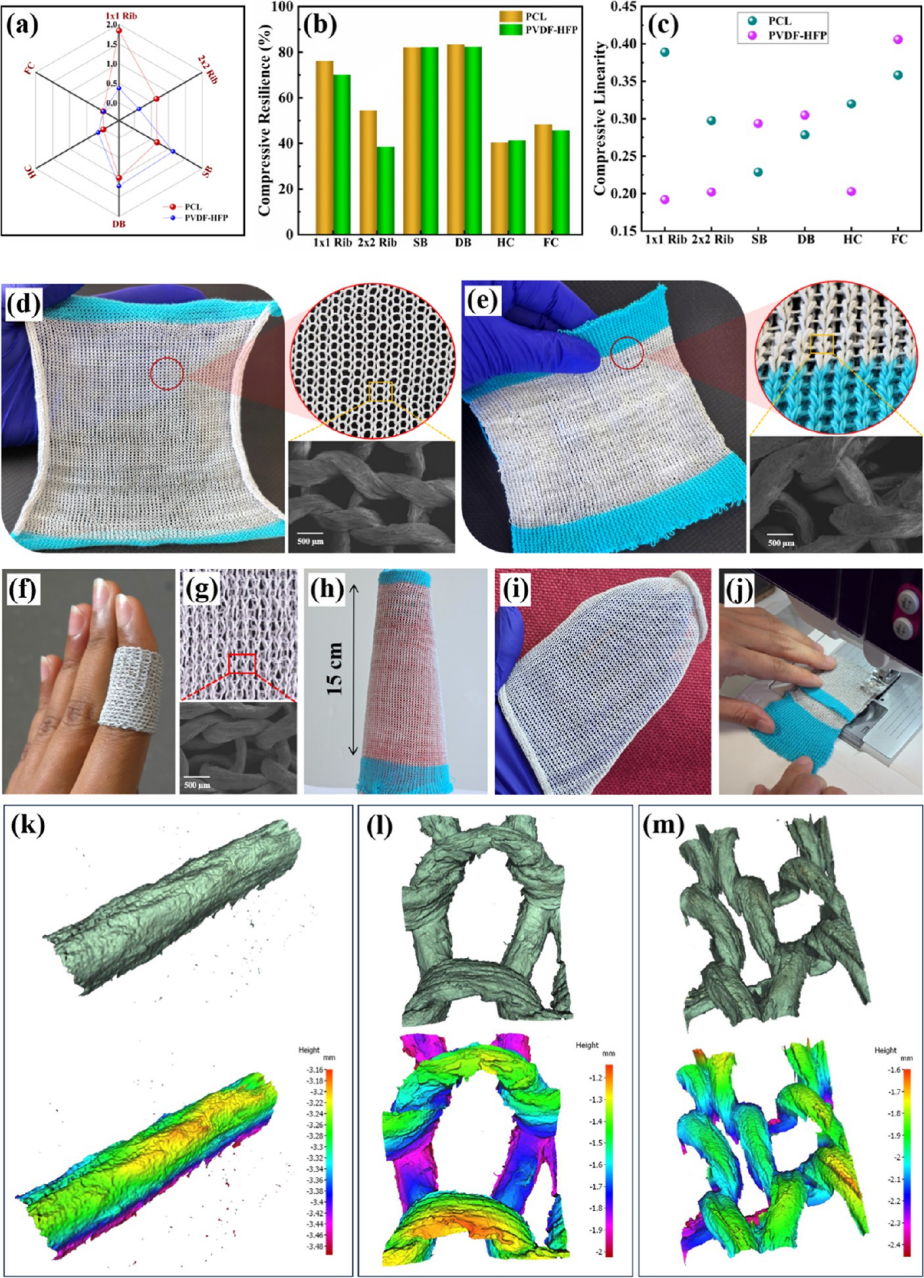
Fabric compression recovery performance of NF with various knitted structures created using PCL and PVDF-HFP CSNY: (a) compressive energy, (b) compressive resilience, and (c) compressive linearity. Photograph of the PCL-based wearable NFs: (d) SB NF (blue yarn being a commercial waste yarn used during the knitting process) and the inset showing an enlarged image displaying the knitting pattern and the SEM image and (e) DB NF (blue yarn being a commercial waste yarn used during the knitting process) and the inset showing an enlarged image displaying the interlocked knitting pattern and the SEM image. PVDF-HFP-based wearable NF: (f) photograph of the DB NF and (g) enlarged image (inset shows the SEM image of the DB structure). (h) Photograph of the knitted fabric formed around a yarn-winding cone and (i) covering a hand. (j) Stitching two DB NFs together using a standard sewing machine. The 3D surface profile of (k) CSNY, (l) SB NF, and (m) DB NF.

In garment production, fabric sewability is essential for forming larger, 3D shaped garments. To evaluate the sewability of the NF, two DB NF knitted samples, with dimensions 5 × 6 cm^2^ and 3 × 5 cm^2^, were sewn together using a standard lockstitch sewing machine (Figure 6j). Following the stitching process, the NF maintained its structural integrity, as demonstrated in Video S3. Three-dimensional (3D) surface profilometric analysis was performed to evaluate the surface texture and irregularities of *C*-yarn, CSNY, and NFs. Figure 6k–m displays the 3D surface profiles of the PCL CSNY, SB, and DB NFs. The line roughness of the *C*-yarn and the PCL CSNY are shown in Figure S10.

Afterward, the extension of SB and DB NFs was measured and analyzed along their wale and course direction. Extensibility indicates the ability of the fabric to extend under different loads.^36^ The percentages of extension of SB and DB fabric under 4.9, 19.6, and 98.1 N/m (5, 20, and 100 gf/cm, respectively) in the course and wale direction are shown in Figure 7a. The elongation of DB fabric in the course direction under 19.6 N/m load exceeded the upper limit of the measurement range of the FAST extension meter, indicating that the fabric has high extensibility. Then, the tensile mechanical properties of SB and DB were also determined along the wale and course direction, and the results are shown in Figure 7b–e. The force–elongation curves show that the NF exhibits anisotropic mechanical properties, as shown in Figure 7 (b). As expected, the course direction demonstrated superior elongation, with the DB NF exhibiting a high elongation of 261.8% along the course direction, as the loops can more easily extend and deform (Figure 7c). However, DB NF along the wale direction demonstrated higher breaking force, tensile strength, and young’s modulus due to the structural integrity and the vertical alignment of the interlinked loops and the reduced stretchability in this direction from loop locking, which makes the fabric more resistant to breaking or deforming when subjected to tensile forces. Statistical analysis showed that the breaking load of DB NF along the wale and course direction was 350.5 and 306.1 N, respectively. The breaking load was lower for SB NF, 139.3 N in the wale direction and 125.4 N in the course direction, as shown in Figure 7d. DB exhibited a maximum tensile strength of 17.53 MPa and Young’s modulus of 209.7 MPa along the wale direction, as shown in Figure 7d, and e, respectively. All these results are typical values within the range of commercial conventional materials, which proves the suitability of NYs and fabrics not only for specialist but conventional uses also.

**Figure 7:**
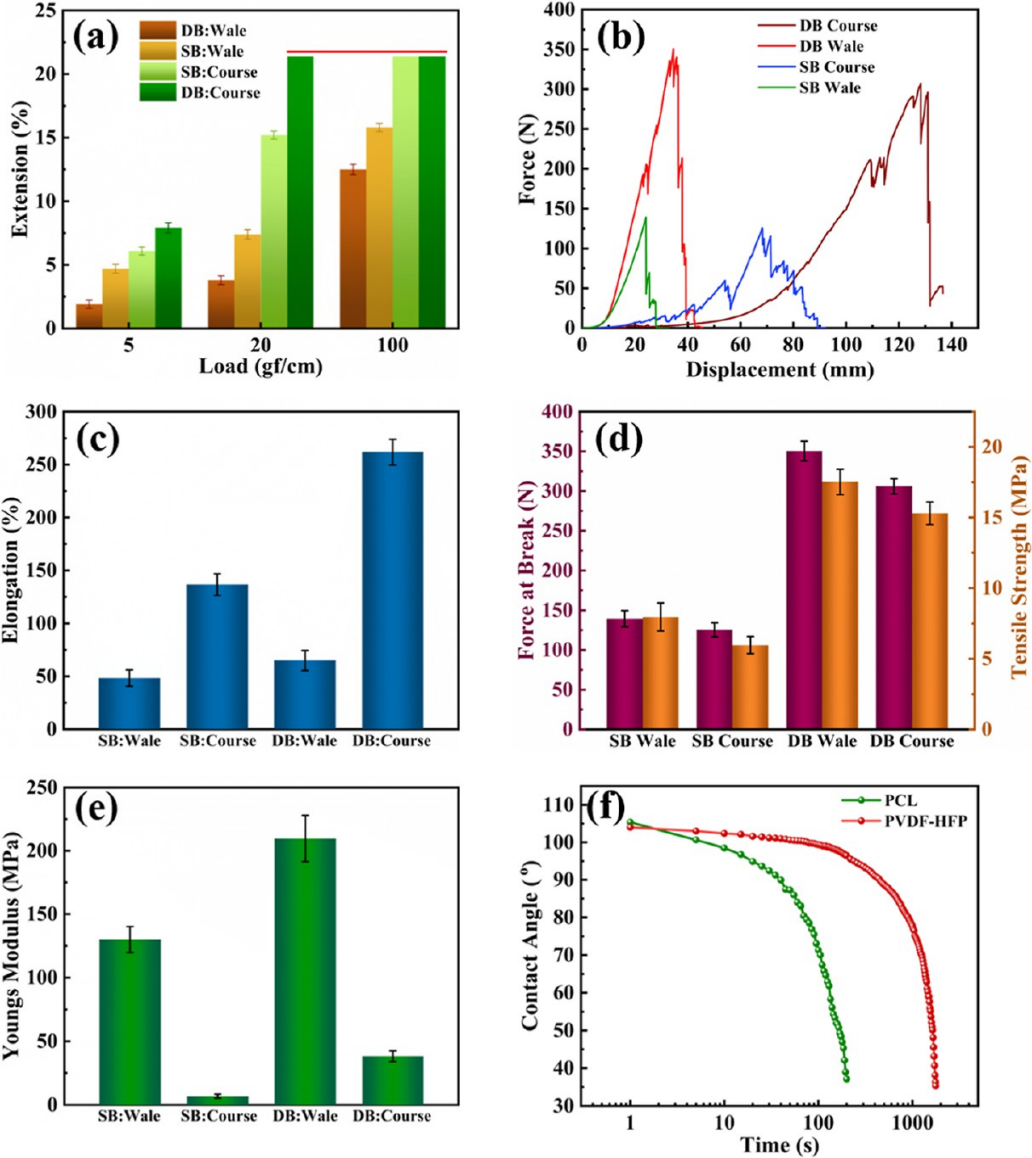
Fabric mechanical properties of SB and DB NF along wale and course directions. (a) Percentage of extension under various loads at 5, 20, and 100 gf/cm. (b) Force-elongation curve, (c) Elongation, (d) Force at break and tensile strength, and (e) Young’s modulus. (f) Water contact angle measurement of the NF at different time intervals.

Testing the wettability and washability of the NF is essential for assessing its surface properties, durability, and functional performance in real-world applications. The wettability of fabrics is typically affected by factors such as surface chemistry, surface roughness, and porosity.^37^ Figure 7f shows the average contact angle of the PCL and PVDF-HFP NF measured at different time intervals. Initially, the PCL NF showed an average contact angle of 105.38°, which was reduced to 46.9° after 3 min of exposure, indicating that its nanofibrous sheath initially resists water but gradually starts to absorb more water. In contrast, the water contact angle for PVDF-HFP NF decreases from 104.04° to 35.3°, over a much longer period of 29 min, indicating absorbing water but at a slower rate than PCL. PVDF-HFP nanofibers are more hydrophobic than PCL nanofibers due to the presence of fluorine in their chemical structure, which significantly enhances water repellence.^38^ Hence, as in conventional fabrics, we can tailor the water properties of NFs by selecting different polymers to meet specific needs. One of the critical factors influencing the usefulness, practicality, longevity, and usability of a fabric is its washability. Typical garments made of fabrics are subject to regular use, which leads to the accumulation of sweat, dirt, and other contaminants. The ability to withstand frequent washing without degrading their quality or functionality is essential for the durability of fabrics.

To test the washability of these NFs, they were subjected to a washing process in an aqueous detergent solution for 10 min, as shown in Figure S11a (Video S4). Figure S11b,c displays the photograph of SB and DB, respectively, after washing and drying. The nanofibrous sheath remained stable after the washing process, demonstrating the good washability and resilience of the fabric, demonstrating the suitability of these fabrics for garment wearing, and leapfrogging textile manufacture to a nanotextiles era. In this study, for the first time, we made durable NFs capable of commercial uses. We examined the chemical, structural, mechanical, and wearable properties of those fabrics, which reveal their superiority when compared with those reported in existing literature, as summarized in Table S1.

### 3.4 Electrical Conductivity and Energy-Harvesting Performance of NF-TENG

Prior to the use of CSNYs in a triboelectric fabric, it is crucial to assess their electrical conductivity and pathway to facilitate efficient charge collection and transport, thereby enhancing both energy generation and operational stability. The developed CSNYs-based NF, featuring a Ag core yarn with a resistance of 300 Ω/m, functions as an effective conductive pathway, much like traditional electric wires embedded within fabric structures. This unique property qualifies it as an “Electronic-NF’’ (*E*-NF). Using this *E*-NF, we developed a range of smart wearable electronics, such as gloves, wristbands, knee bands, and socks, designed for various applications, as shown in Figure 8a. These include human-machine interfacing, health monitoring (such as tracking blood pressure, sleep, and gait), and sensing (pressure, gas, sweat, temperature, and humidity). Additionally, the NF can function as an energy device, harnessing biomechanical energy and storing it. To validate the integrity of its electrical pathways, we conducted charging experiments with external capacitors connected to a direct current (DC) voltage source through the NF. The results demonstrated that the NF supports efficient electron flow and maintains stability even after the knitting process, confirming its structural and functional durability for practical applications. Our findings highlight a clear relationship between the surface area of the NF and the charging speed of various capacitor sizes, such as 4.7 μF, 10 μF, and 47 μF. Smaller configurations, such as the 18 cm long CSNY, consistently exhibited faster charging times compared to larger than 15 cm^2^ NF area configurations. This pattern of results aligns with Ohm’s Law, which indicates that as the fabric area increases, the effective path length for electron flow also increases, resulting in higher resistance and consequently slower charging times. The effect of surface area on charge transfer efficiency can be further explained by the relationship

5 $$R=\rho \frac{L}{A}$$

where resistance *R* increases with path length *L*. The charging times clearly show that the CSNY configuration achieves the fastest charge rates across all capacitance values, taking only 2 ms to charge a 4.7 μF capacitor, in contrast to 30.5 ms required by the larger fabric. As fabric area increases, the time required to charge capacitors grows significantly; for instance, the large fabric configuration requires 255.5 ms to charge a 47 μF capacitor. This extended charging time indicates a trade-off: while larger fabrics support higher capacitance, they exhibit a slower charging rate, which may be beneficial in applications that require sustained power output rather than quick pulses. Figure 8b–d shows the voltage-time response for different capacitor configurations connected to a CSNY with a length of 18 cm, an NF with an area of 3.5 cm^2^ and 15 cm^2^, respectively.

**Figure 8:**
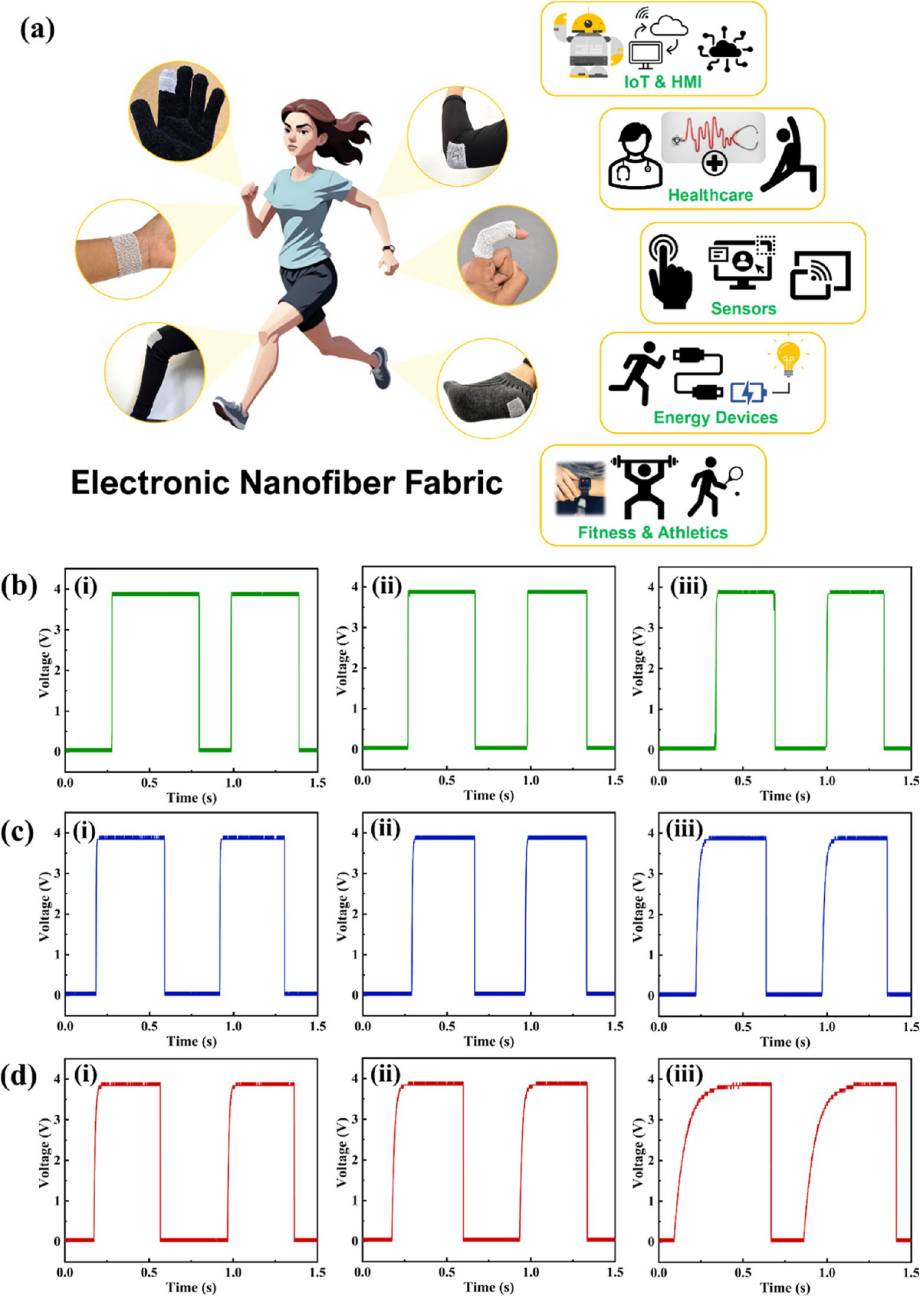
(a) Schematic of smart electronic NF attached to different body parts, all designed for various applications. Voltage-time response of different capacitor configurations ((i) 4.7 μF, (ii) 10 μF, and (iii) 47 μF) connected to (b) CSNT with a length of 18 cm. (c) NF with an area of 3.5 cm^2^ and (d) NF with an area of 15 cm^2^.

To evaluate the energy harvesting efficiency of the NFs structures, an NF-TENG was developed using PCL NF as the tribopositive material and PVDF-HFP NF as the tribonegative material. Under the contact separation mechanism, the developed NF-TENG device with an effective contact area of 4 cm^2^ generated an open circuit voltage of ∼ 100 V and a short circuit current of ∼8 μA, as shown in Figure 9a,b, respectively. Since the NF-TENG generates alternating currents (AC), which are incompatible with most small electronic devices that require DC, a full-wave bridge rectifier circuit was integrated to convert the generated AC into DC. The rectified output signals, displayed in Figure 9c,d, demonstrate the feasibility of the NF-TENG for energy storage applications, such as capacitor charging. The capacitor charging profile of the NF-TENG tested with various commercial capacitors (1, 4.7, 10, and 47 μF) over a 50 s interval is presented in Figure 9f. Notably, a 1 μF capacitor achieved a saturation voltage of 3 V within 20 s, while higher capacitance values resulted in proportionally lower saturation voltages. These results highlight the high energy harvesting potential of the NF-TENG, demonstrating its ability to power small electronic devices and paving the way for self-powered electronic applications.

**Figure 9:**
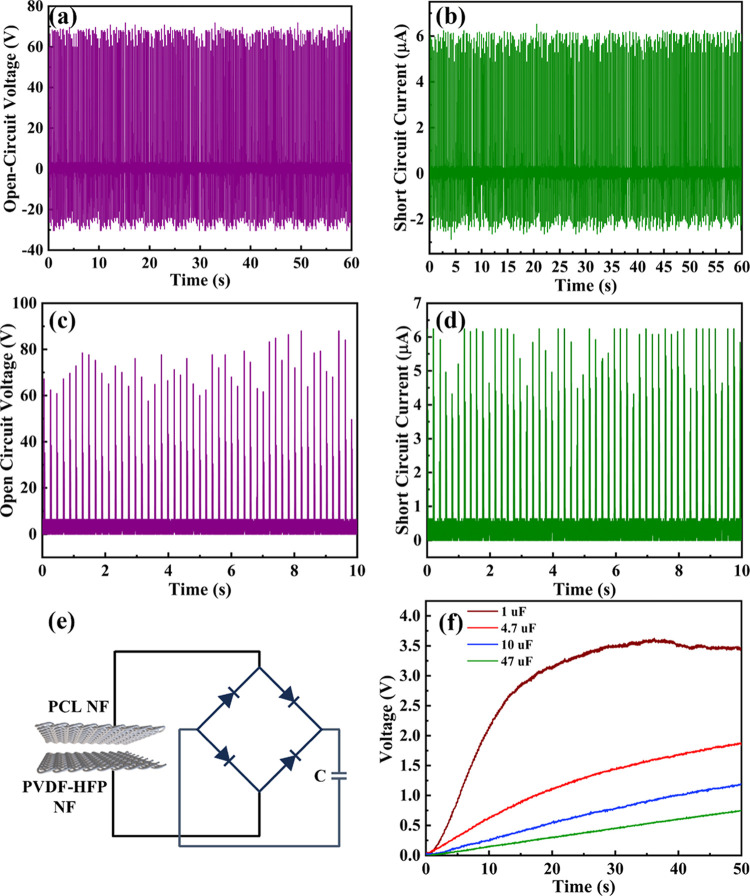
PCL/PVDF-HFP NF based TENG. (a) Open circuit voltage and (b) short circuit current generated by NF-TENG. The rectified output performance of NF-TENG: (c) rectified voltage and (d) rectified current. (e) Full-wave bridge rectifier circuit for capacitor charging. (f) Capacitor charging profile of the NF-TENG.

## 4 Conclusion

This study presents a significant breakthrough in the scalable fabrication of CSNYs using the NanoTwist Spinning technique, which is a novel and industrially viable approach for developing high-performance energy-harvesting NFs. By integrating electrospinning and yarn-twisting, we successfully engineered durable, flexible, and knittable CSNYs composed of a nanofiber sheath uniformly wrapped around a conductive core, enabling seamless processing into wearable electronic textiles (E-textiles). Our optimized with PCL- and PVDF-HFP-based CSNYs demonstrated remarkable mechanical resilience, with an 83% compressive recovery rate, a tensile strength of 17.53 MPa, and an elongation of 261.8%, ensuring their long-term wearability and robustness in textile applications. Furthermore, the knitted NFs exhibited excellent washability, flexibility, and compatibility with industrial textile manufacturing, addressing a key challenge in the commercialization of nanofiber-based textiles. Beyond mechanical performance, the electrical and triboelectric properties of these NFs were evaluated in an NF-TENG, achieving a high electrical output of 100 V and 8 μA under real-world conditions. These findings establish CSNY-based NFs as a promising platform for self-powered smart textiles capable of harvesting biomechanical energy from human motion for wearable electronics, health monitoring, and next-generation e-textiles. This research not only advances the scalability and practicality of energy-harvesting textiles but also opens up exciting possibilities for their integration into smart clothing, biomedical sensors, and electronic textiles. Future work will focus on further optimizing the electrospinning-yarn twisting process, enhancing energy conversion efficiency, and exploring real-world deployment scenarios for next-generation wearable energy systems.

## Supplementary Material











## Notes

Fabrication of nanofiber yarn using combined nanospinning, yarn twisting, and collecting process (MP4)
Production of knitted NF (MP4)
Stitching NF using an industrial sewing machine (MP4)
Washing test of NF (MP4)
Selection of *C*-yarn, optimization of electrospinning parameters (voltage optimization, flow rate and needle distance optimization, and take-up speed optimization), embroidery designs created using PCL CSNYs, compression testing, PCL CSNY-based knitted NF, 3D optical surface measurements, washability test, electrical resistance of conductive yarn under stretching, comparison of the properties of previously reported CS NFs for various wearable applications with this work, and comparison of the output performance of previously reported contact separation mode NF-TENGs with this present work (PDF)
