# Supplementary material for: Scalable Fabrication of Core–Sheath Nanofiber Yarns via NanoTwist Spinning for High-Performance Energy-Harvesting E‑Nanofiber Fabrics
Source: ACS Appl Mater Interfaces. 2025 Jun 17;17(26):37936–50. doi: 10.1021/acsami.5c04482 (PMC12232280; doi:10.1021/acsami.5c04482)
Supplement: Supplementary file 5 [file am5c04482_si_005.pdf]

SUPPORTING INFORMATION

Scalable Fabrication of Core-Sheath Nanofiber Yarns via NanoTwist Spinning for High-Performance Energy-Harvesting E-Nanofiber Fabrics

Syamini Jayadevan, Akshaya Kumar Aliyana, and George K Stylios*

Smart Wearable Electronics Group (SWEG), Research Institute for Flexible Materials, School of Textiles and Design, Heriot-Watt University, UK

*g.stylios@hw.ac.uk

Selection of C-yarn

In the PCL CSNY, the core component is a commercial conducting yarn. The selection of this yarn is based on criteria such as mechanical strength, conductivity, and flexibility. First, the mechanical properties of the yarns were tested, with samples 5 and 7 showing superior mechanical properties (Fig. S1 (a)). Secondly, conductivity tests revealed that samples 3, 4, and 5 had the highest conductivity due to their low resistivity (Fig. S1 (b)). Thirdly, in terms of flexibility and wearability, samples 2, 3, 4, and 5, being metallic monofilament yarns, lacked flexibility. Considering all these parameters, sample 7 seems to be the ideal choice due to its superior mechanical properties and excellent flexibility and wearability with acceptable conductivity.

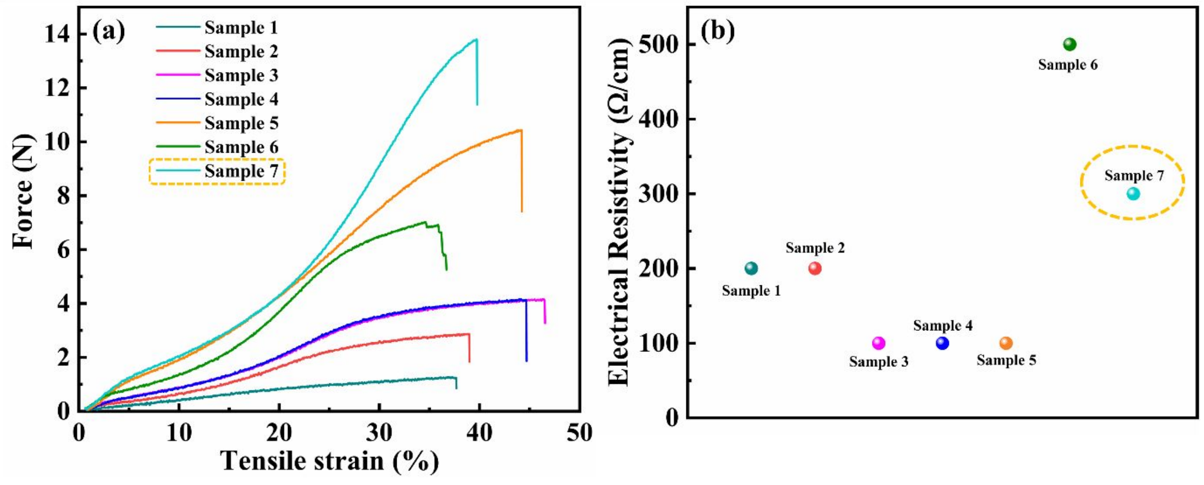

Fig. S1. (a) The tensile testing and (b) electrical resistance of various conducting C-yarns.

Optimization of electrospinning parameters

1. Voltage optimization

The effect of applied voltage on the formation of PCL nanofibers was analyzed by varying the voltage between 7 and 14 kV. The SEM images of PCL-based CSNYs fabricated at different voltages are shown in Fig. S2. At 7 kV, nanofiber formation was initiated, however, the nanofiber deposition was minimal, due to unstable jet formation, and frequent clogging at the spinneret. These issues are attributed to insufficient electrostatic force to overcome the surface tension of the polymer solution, leading to irregular and incomplete fiber formation. At an applied voltage of 9 kV, the nanofiber formation improved significantly. Uniform fibers were observed, and consistent nanofiber wrapping occurred around the core yarn. The nanofiber diameters ranged from 400 to 1500 nm, with an average diameter of 850 ± 14 nm. This voltage provided an optimal balance between electrostatic force and solution flow rate, promoting stable jet formation and uniform fiber deposition. When the voltage was increased to 10 kV, the wrapping remained uniform, but the fiber diameter distribution became broader, ranging from 300 to 2000 nm, with an average diameter of 1038 ± 38 nm. This can be attributed to the higher stretching forces acting on the jet, leading to irregular thinning and the formation of both thinner and thicker fibers. As the applied voltage was further increased to the range of 11–14 kV, an insufficient nanofiber wrapping was observed, as shown in Fig. S2 (h), where the core yarn is prominently visible due to minimal nanofiber coverage.

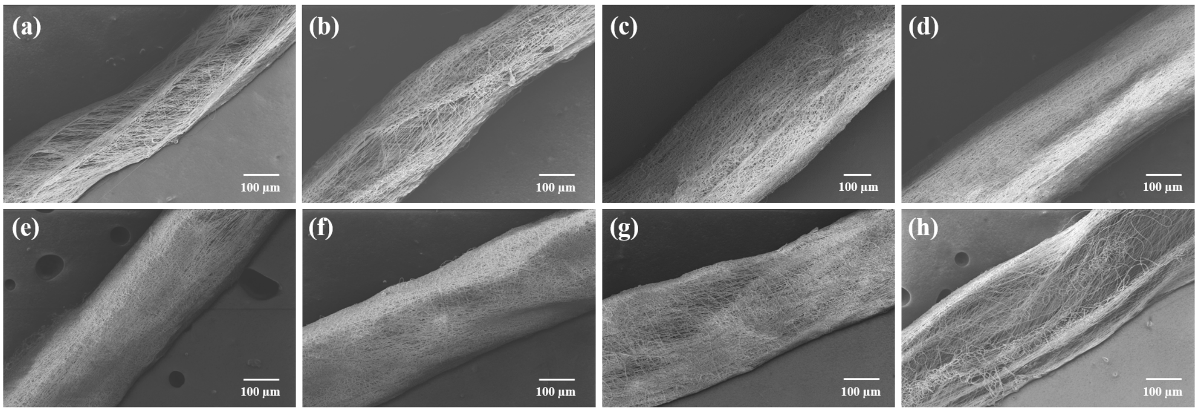

Fig. S2. SEM image of PCL CSNY fabricated by varying the applied voltage: (a) 7 kV, (b) 8 kV, (c) 9 kV, (d) 10 kV, (e) 11 kV, (f) 12 kV, (g) 13 kV, and (h) 14 kV.

2. Flow rate and needle distance optimization

The distance of the spinneret from the funnel collector and the flow rate of the polymer solution through the spinneret influences the morphology of the nanofibers. An optimum needle distance and flow rate needs to be maintained to prepare smooth and uniform nanofibers. This critical value varies with the polymer system. To investigate the effect of needle distance and flow rate, 15 wt.% PCL was electrospun from three different needle distance (10, 11, and 12 cm away from the funnel) with four flow rates (0.5, 0.7, 1, and 1.5 mL/h). Fig. S3 and S4 shows the diameter distribution of PCL nanofibers at different needle distance and solution flow rate.

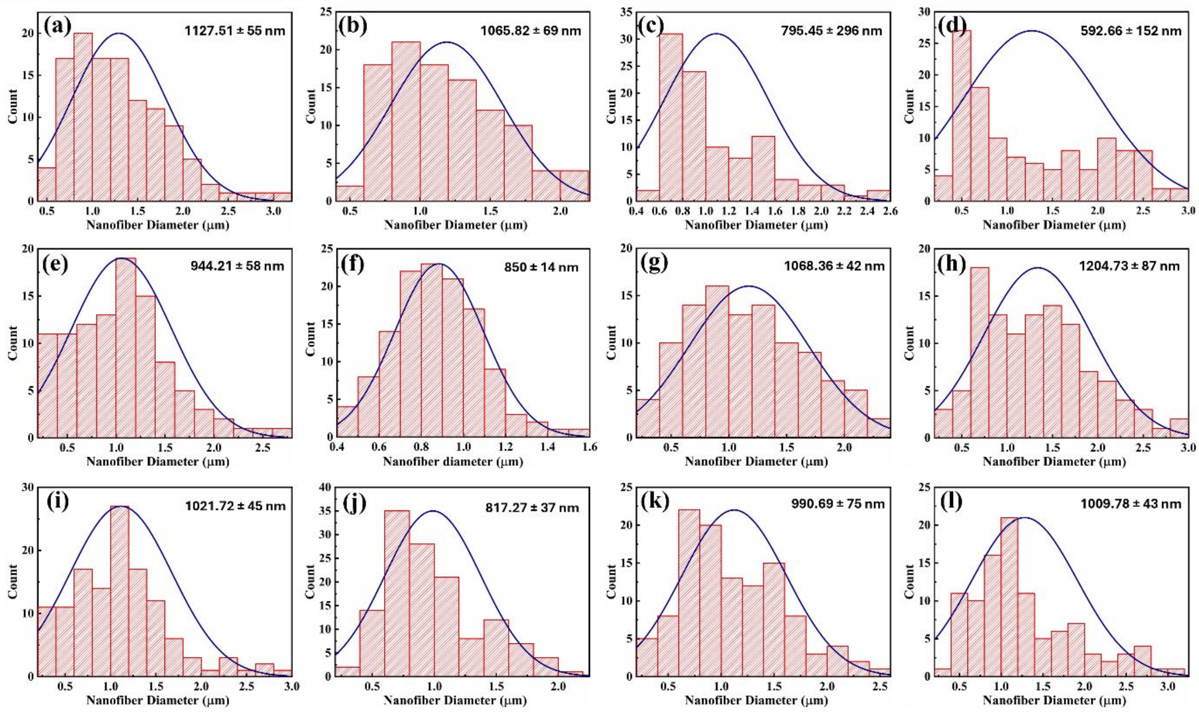

Fig. S3. The diameter distribution of PCL nanofibers at different needle distance and solution flow rate. Needle distance 10 cm and varying solution flow rate: (a) 0.5 mL/h, (b) 0.7 mL/h, (c) 1 mL/h, and (d) 1.5 mL/h. Needle distance 11 cm and varying solution flow rate: (e) 0.5 mL/h, (f) 0.7 mL/h, (g) 1 mL/h, and (h) 1.5 mL/h. Needle distance 12 cm and varying solution flow rate: (i) 0.5 mL/h, (j) 0.7 mL/h, (k) 1 mL/h, and (l) 1.5 mL/h.

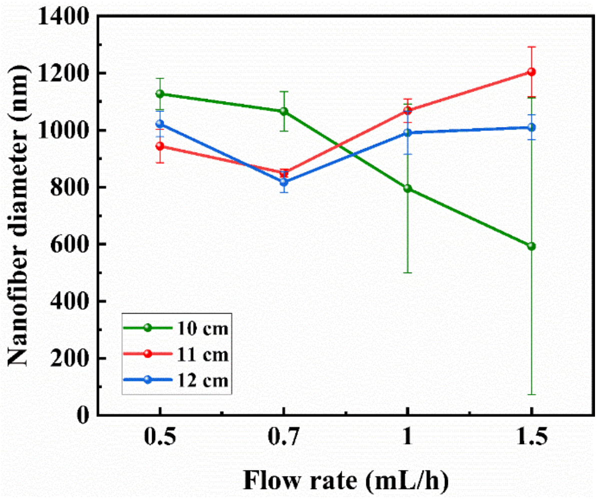

Fig. S4. Variation of average PCL nanofiber diameter at different flow rate and needle distance.

The SEM images of the PCL CSNY fabricated by varying the needle distance and the solution flow is shown in Fig. S5. The optimum PCL CSNY with uniform wrapping and finer nanofiber diameter was obtained by adjusting these parameters.

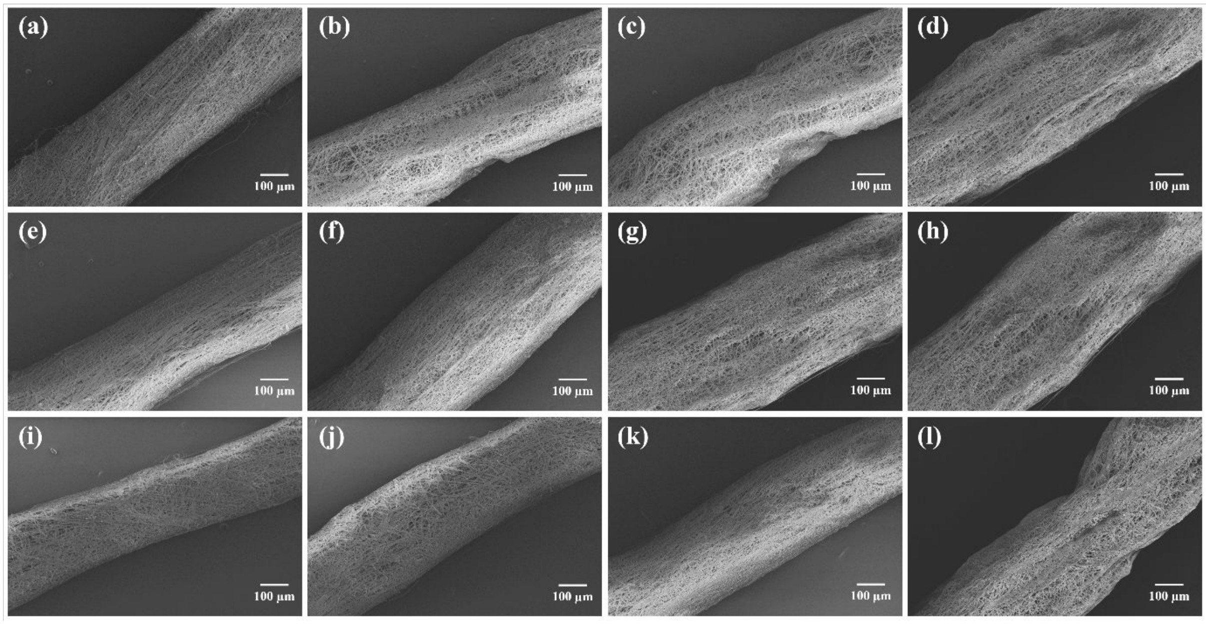

Fig. S5. SEM image of PCL CSNY fabricated by varying the solution flow rate and needle distance. Needle distance 10 cm and varying solution flow rate: (a) 0.5 mL/h, (b) 0.7 mL/h, (c) 1 mL/h, and (d) 1.5 mL/h. Needle distance 11 cm and varying solution flow rate: (e) 0.5 mL/h, (f) 0.7 mL/h, (g) 1 mL/h, and (h) 1.5 mL/h. Needle distance 12 cm and varying solution flow rate: (i) 0.5 mL/h, (j) 0.7 mL/h, (k) 1 mL/h, and (l) 1.5 mL/h.

3. Take-up speed optimization

The thickness of the PCL CSNY can be controlled by adjusting the take-up speed. Increasing the take-up speed decreases the wrapping time of the nanofibers around the C-yarn, leading to higher productivity but resulting in a reduced yarn diameter and thinner nanofiber sheath. The SEM images of the PCL CSNYs collected at different take-up speed is shown in Fig. S6.

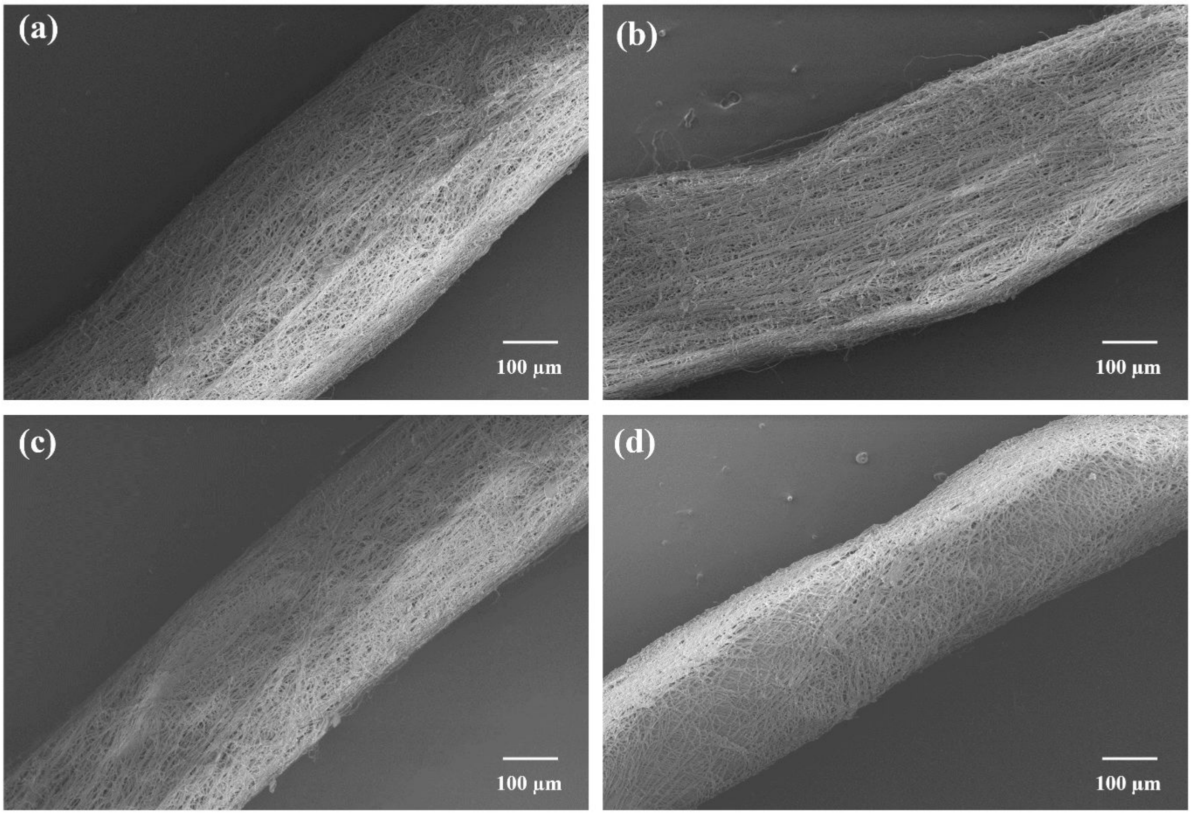

Fig. S6. SEM image of PCL CSNY collected at different take-up speed: (a) 0.8 rpm, (b) 1 rpm, (c) 1.2 rpm, and (1.4) rpm.

Embroidery designs created using PCL CSNYs

Various hand embroidery stitches, knots, and patterns were crafted using the PCL CSNY on a black cotton fabric, as shown in Fig. S7. The CSNY exhibited excellent suitability for embroidery applications, demonstrating its ability to create durable designs for decorative and smart applications.

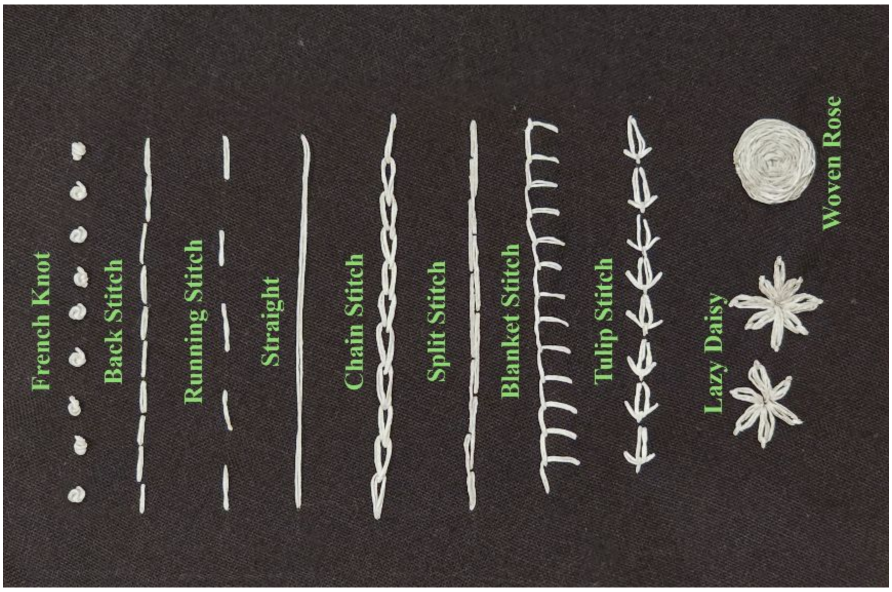

Fig. S7. Various embroidery stitches, knots and patterns were crafted using the PCL CSNY.

Compression Testing

The photograph captured during the compression test is shown in Fig. S8 (a), and a typical compression recovery curve is shown in Fig. S8 (b).

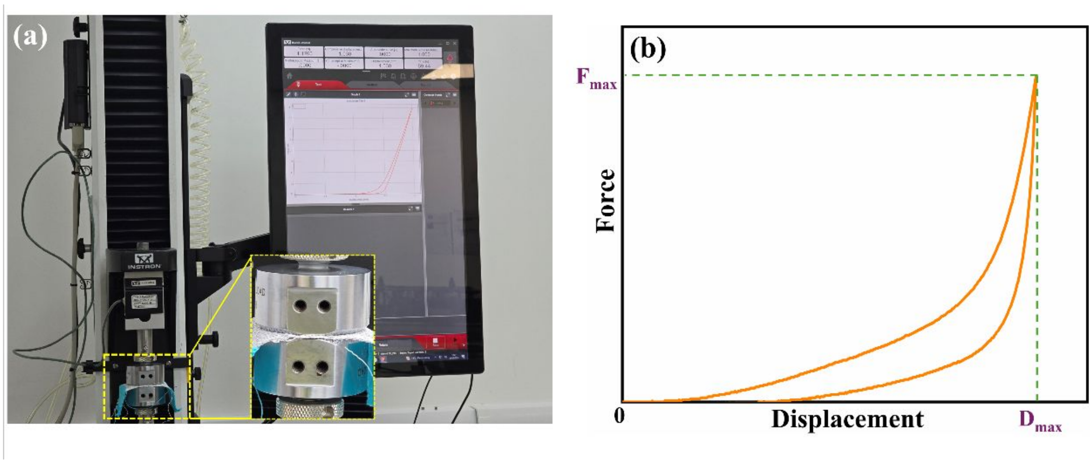

Fig. S8. (a) Fabric compression performance test setup. (b) Fabric compression recovery curve.

PCL CSNY-based knitted NF

The dimensions of the PCL CSNY-based SB and DB NFs are shown in Fig. S9 (a, b) (with the blue yarn being a commercial yarn used during the knitting process). The SB NF measures 16 cm in length and 15 cm in width, while the DB NF measures 7 cm in length and 11.5 cm in width. Their exceptional flexibility is demonstrated by folding the NFs (Fig. S9 (c-f)).

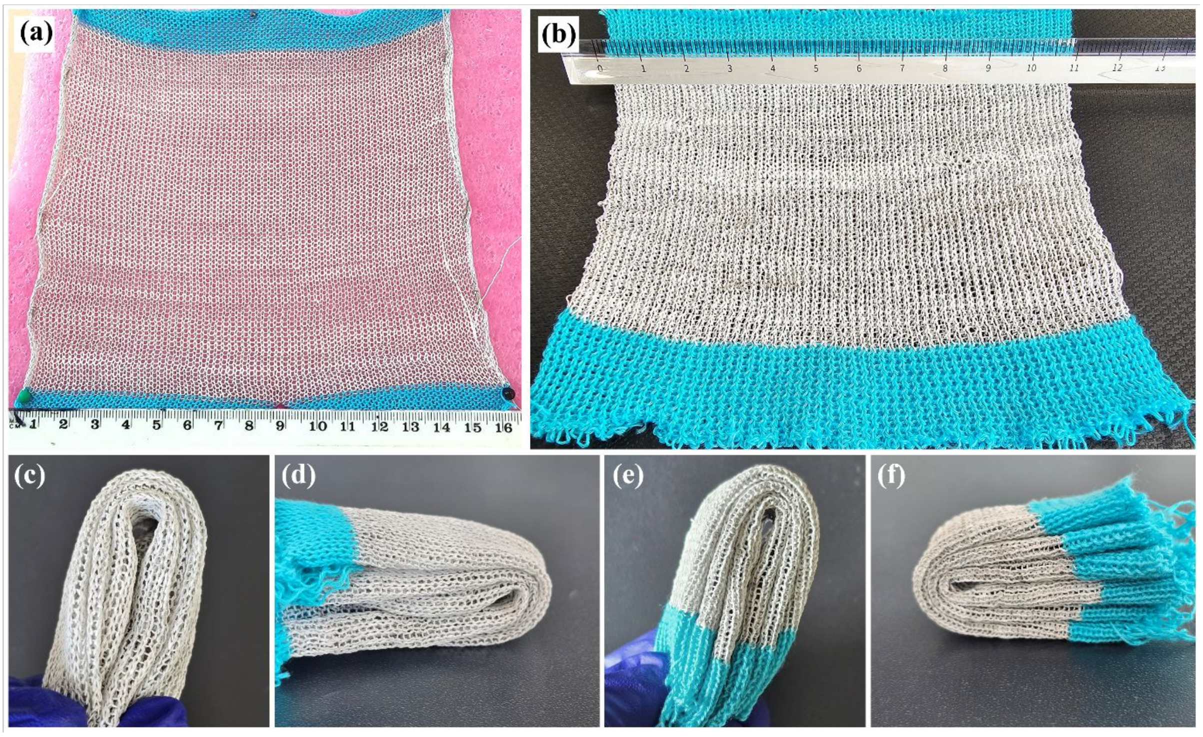

Fig. S9. PCL CSNY-based NFs: (a) SB and (b) DB NFs. Foldability of the NFs: (c, d) SB and (e, f) DB NFs.

3D Optical Surface Measurements

The surface 3D profile of the PCL CSNY and the C-yarn were analyzed, and their respective line roughness is shown in Fig. S10.

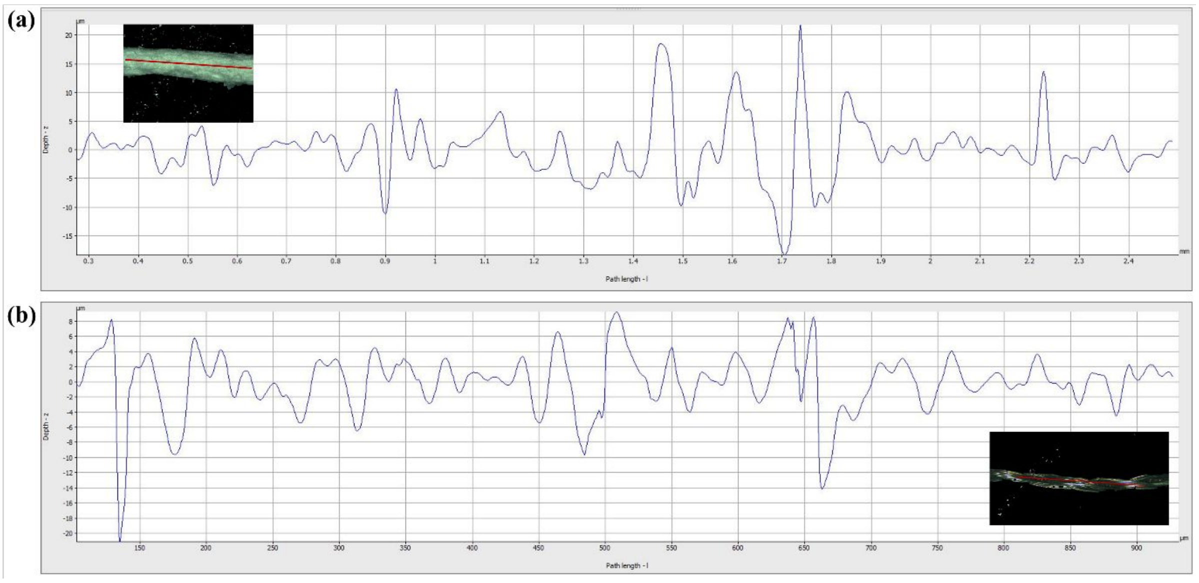

Fig. S10. The line roughness of (a) PCL CSNY and (b) C-yarn.

Washability Test

The SB and DB NFs were subjected to a washing process in an aqueous detergent solution for 10 min, as shown in Fig. S11 (a). Fig. S11 (b) and (c) display the photograph of the NFs after washing and drying. The SEM images of SB and DB NFs before and after washing are shown in Fig. S11 (d-g). The nanofibrous sheath remained stable after the washing process, demonstrating the stability, resilience, and washability of the NFs.

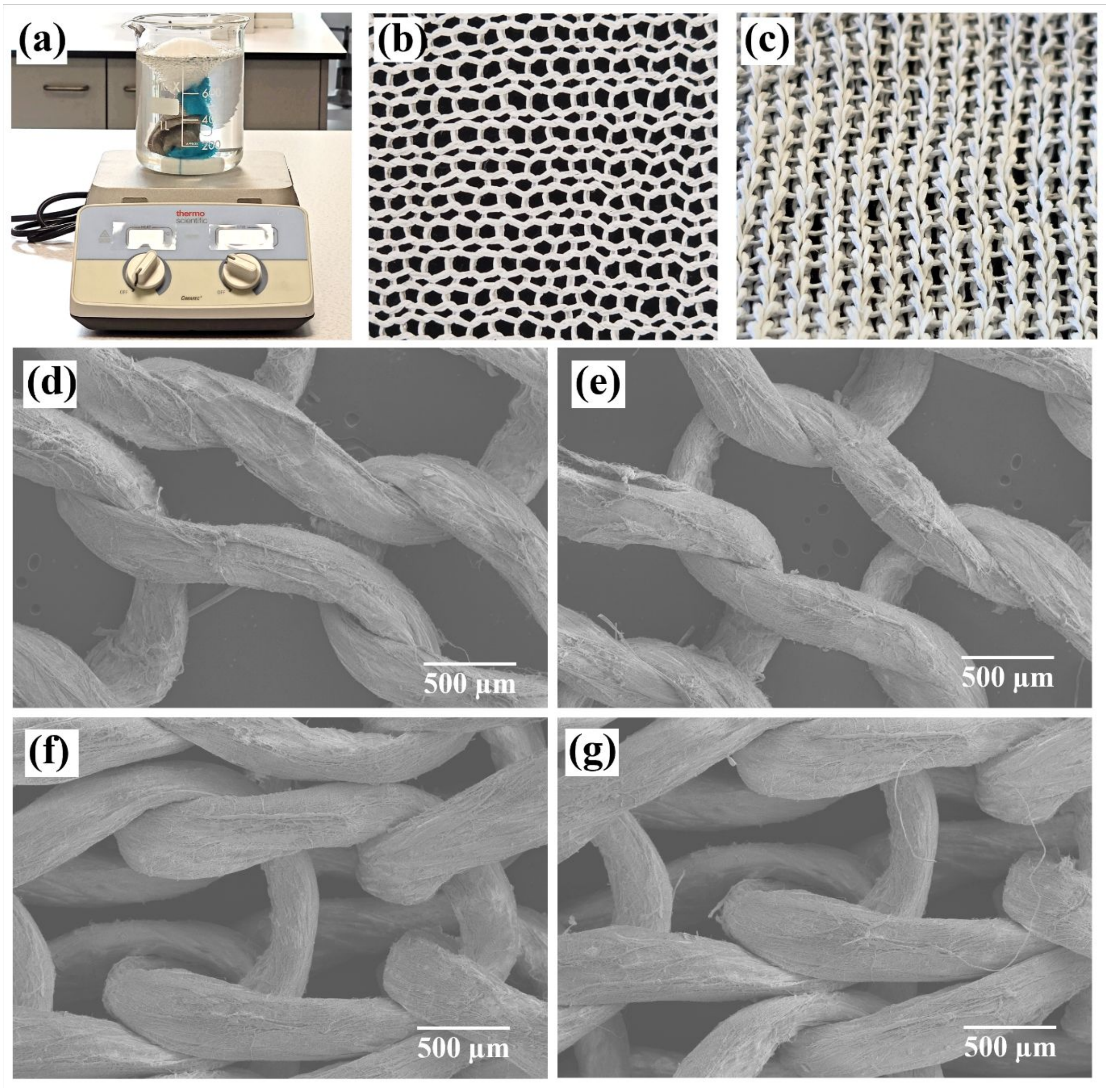

Fig. S11. (a) Washability test of knitted NFs using a magnetic stirrer. Photograph of the NFs after washing: (b) SB and (c) DB NFs. SEM image of the SB fabric: (d) before and (e) after washing. SEM image of the DB fabric: (d) before and (e) after washing.

Electrical Resistance of Conductive Yarn under Stretching

An 8 cm long C-yarn was used to investigate the effect of mechanical stretching on its electrical resistance. The graph shows that the electrical resistance of the conductive yarn increases gradually with displacement (stretching) and then rises sharply at higher strain levels. Up to 20 mm of stretching, the conductive pathways remain relatively stable, resulting in a moderate and linear resistance increase. The rapid increase in the resistance beyond 20 mm stretching can be attributed to the structural deformation and disruption of the conductive network. Since typical human body movements induce only small strains, usually much less than 20 mm, the yarn is well-suited for smart wearable applications, as it can maintain a stable electrical performance under normal mechanical deformations.

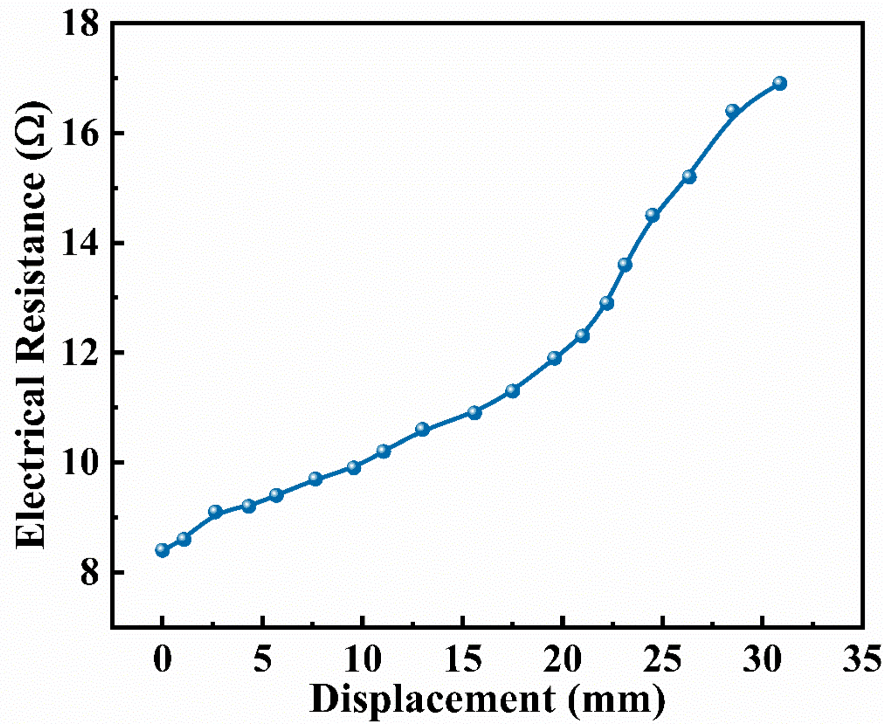

Fig. S12. Effect of mechanical stretching on the electrical resistance of C-yarn

Table S1. Comparison of the Properties of Previously Reported CS NFs for Various Wearable Applications with this Work

**Core**	**Nanofiber Sheath**	**Fabric developm ent**	**Fabric dimension**	**Wearable application**	**Tensile strength**	**Fabric behavior and wearability**	**Washab ility test**	**Ref**
Ag	PVDF/PAN	Home-made weaving	~2.5×2.5 cm^2	Energy harvesting and sensing	Tested	-	-	[1]
Stainless steel	PVDF	Stitching	-	Energy harvesting and sensing	Tested	-	-	[2]
Stainless steel	PVDF and PA66	Hand weaving, and knitting	-	Energy harvesting and sensing	Tested	-	-	[3]
Cu fibers	PLLA	Home-made weaving	4×4 cm^2	Energy harvesting	Tested	-	-	[4]
Cotton	GO-PEI-Ag/PAN	Weaving	-	Antibacteria l fabric	-	-	Tested	[5]
Ag/nylon	PVDF-TrFE	Home-made weaving	4×4 cm^2	Energy harvesting and sensing	Tested	-	Tested	[6]
Cu	PVDF-TrFE	Home-made weaving	4×4 cm^2	Energy harvesting and sensing	Tested	-	Tested	[7]
Nickel-coated cotton	PU	Home-made weaving	5×5 cm^2	Pressure sensor	-	-	-	[8]
Ag/nylon	PVDF/PTF E and PCL	Weaving	3×3 cm^2	Energy harvesting and self-powered electronics	-	-	-	[9]
**Ag/Nylon**	**PCL and PVDF-HFP**	**Industrial knitting**	**16×15 cm^2 (for SB) 11.5×7 cm^2 (DB)**	**-**	**Tested**	**Tested**	**Tested**	**This work**

Table S2. Comparison of the Output Performance of Previously Reported Contact Separation Mode NF-TENGs as with This Present Work

Triboelectric Materials	Applied Force (N)	Frequency (Hz)	Area of the device (cm^2)	Output Performance		Ref
				Voltage (V)	Current (μA)	
PA – PVDF woven NF	0.6 kPa	3.5	-	12	0.08	[3]
PVDF-TrFE woven NF,<br>Nylon fabric (commercial)	35	1.2	8	100	2.5	[6]
PCL woven NF,<br>PVDF/PTFE woven NF	37.5	5	9	20	2.26	[9]
Rubber film,<br>PVDF-TrFE/PA woven NF	200	5	25	87	9	[10]
PU woven NF,<br>Si_3N_4/ PVDF PU woven NF	-	3 Hz	25	71	0.715	[11]
PI/SiO_2 – PTFE woven NF	50	1 Hz	25	30	0.45	[12]
Skin,<br>PVDF woven NF	6	3	64	250	1.75	[13]
PA fabric (commercial),<br>PI/MXene woven NF	25	3	9	138	9.3	[14]
Nylon fabric (commercial),<br>PI/PVDF-PDMS knitted NF	235 kPa	1	16	14	0.86	[15]
Silk fabric (commercial),<br>PVDF/Graphene woven NF	28 kPa	5	25	87.3	0.281	[16]
Acrylic sheet,<br>F-PI	50	2	4	75.3	0.27	[17]
Bacterial Cellulose/PVA woven NF,<br>PDMS film coated commercial fabric	10	5	1.5	150	3.8	[18]
PA66 nanofiber mat,<br>PTFE knitted NF	20	3	15	200	16	[19]
**PCL knitted NF,<br>PVDF-HFP knitted NF**	**8**	**4**	**4**	**109**	**6.9**	**This Work**

Supplementary Video 1: Fabrication of nanofiber yarn using combined nanospinning, yarn twisting, and collecting process

Supplementary Video 2: Production of knitted NF

Supplementary Video 3: Stitching NF using an industrial sewing machine

Supplementary Video 4: Washing test of NF

References

[1] L. Ma, M. Zhou, R. Wu, A. Patil, H. Gong, S. Zhu, T. Wang, Y. Zhang, S. Shen, K. Dong, L. Yang, J. Wang, W. Guo, Z.L. Wang, Continuous and Scalable Manufacture of Hybridized Nano-Micro Triboelectric Yarns for Energy Harvesting and Signal Sensing, ACS Nano 14 (2020) 4716–4726. https://doi.org/10.1021/acsnano.0c00524.

[2] C. Ye, S. Yang, J. Ren, S. Dong, L. Cao, Y. Pei, S. Ling, Electroassisted Core-Spun Triboelectric Nanogenerator Fabrics for IntelliSense and Artificial Intelligence Perception, ACS Nano 16 (2022) 4415–4425. https://doi.org/10.1021/acsnano.1c10680.

[3] Y. Wang, L. Chu, S. Meng, M. Yang, Y. Yu, X. Deng, C. Qi, T. Kong, Z. Liu, Scalable and Ultra-Sensitive Nanofibers Coaxial Yarn-Woven Triboelectric Nanogenerator Textile Sensors for Real-Time Gait Analysis, Advanced Science 11 (2024). https://doi.org/10.1002/advs.202401436.

[4] X. Meng, X. Jia, Y. Qi, D. Miao, X. Yan, Fabrication of polylactic acid nanofibrous yarns for piezoelectric fabrics, E-Polymers 23 (2023). https://doi.org/10.1515/epoly-2023-0030.

[5] W. Yu, X. Li, J. He, Y. Chen, L. Qi, P. Yuan, K. Ou, F. Liu, Y. Zhou, X. Qin, Graphene oxide-silver nanocomposites embedded nanofiber core-spun yarns for durable antibacterial textiles, J Colloid Interface Sci 584 (2021) 164–173. https://doi.org/10.1016/j.jcis.2020.09.092.

[6] Y. Chen, J. Hua, Y. Ling, Y. Liu, M. Chen, B. Ju, W. Gao, A. Mills, X. Tao, R. Yin, An airflow-driven system for scalable production of nano-microfiber wrapped triboelectric yarns for wearable applications, Chemical Engineering Journal 477 (2023) 147026. https://doi.org/10.1016/j.cej.2023.147026.

[7] Z. Dai, N. Wang, Y. Yu, Y. Lu, L. Jiang, D.-A. Zhang, X. Wang, X. Yan, Y.-Z. Long, One-Step Preparation of a Core-Spun Cu/P(VDF-TrFE) Nanofibrous Yarn for Wearable Smart Textile to Monitor Human Movement, ACS Appl Mater Interfaces 13 (2021) 44234–44242. https://doi.org/10.1021/acsami.1c10366.

[8] K. Qi, H. Wang, X. You, X. Tao, M. Li, Y. Zhou, Y. Zhang, J. He, W. Shao, S. Cui, Core-sheath nanofiber yarn for textile pressure sensor with high pressure sensitivity and spatial tactile acuity, J Colloid Interface Sci 561 (2020) 93–103. https://doi.org/10.1016/j.jcis.2019.11.059.

[9] M. Zhou, F. Xu, L. Ma, Q. Luo, W. Ma, R. Wang, C. Lan, X. Pu, X. Qin, Continuously fabricated nano/micro aligned fiber based waterproof and breathable fabric triboelectric nanogenerators for self-powered sensing systems, Nano Energy 104 (2022) 107885. https://doi.org/10.1016/j.nanoen.2022.107885.

[10] X. Guan, B. Xu, M. Wu, T. Jing, Y. Yang, Y. Gao, Breathable, washable and wearable woven-structured triboelectric nanogenerators utilizing electrospun nanofibers for biomechanical energy harvesting and self-powered sensing, Nano Energy 80 (2021) 105549. https://doi.org/10.1016/j.nanoen.2020.105549.

[11] X. Tao, Y. Zhou, K. Qi, C. Guo, Y. Dai, J. He, Z. Dai, Wearable textile triboelectric generator based on nanofiber core-spun yarn coupled with electret effect, J Colloid Interface Sci 608 (2022) 2339–2346. https://doi.org/10.1016/j.jcis.2021.10.151.

[12] F. Xing, Z. Ou, X. Gao, B. Chen, Z.L. Wang, Harvesting Electrical Energy from High Temperature Environment by Aerogel Nano-Covered Triboelectric Yarns, Adv Funct Mater 32 (2022). https://doi.org/10.1002/adfm.202205275.

[13] C. Ye, S. Yang, J. Ren, S. Dong, L. Cao, Y. Pei, S. Ling, Electroassisted Core-Spun Triboelectric Nanogenerator Fabrics for IntelliSense and Artificial Intelligence Perception, ACS Nano 16 (2022) 4415–4425. https://doi.org/10.1021/acsnano.1c10680.

[14] J. Yan, H. Wang, K. Wang, W. Kang, G. Yang, Thermally robust hierarchical nanofiber triboelectric yarns for efficient energy harvesting in firefighting E-textiles, Chemical Engineering Journal 499 (2024) 156188. https://doi.org/10.1016/j.cej.2024.156188.

[15] W. Akram, Q. Chen, X. Zhang, S. Ren, L. Niu, J. Fang, Coaxial tribonegative yarn TENG with aromatic polyimide as charge entrapment layer for real-time edge ball

assessment in cricket sports, Nano Energy 131 (2024) 110275. https://doi.org/10.1016/j.nanoen.2024.110275.

[16] T. Yang, C. Wan, X. Zhang, T. Liu, L. Niu, J. Fang, Y. Liu, High-efficiency preparation of multifunctional conjugated electrospun graphene doped PVDF/CF yarns for energy harvesting and human movement monitoring in TENG textile, Nano Res (2023). https://doi.org/10.1007/s12274-023-6373-8.

[17] M. Hao, X. Zhang, H. Pan, X. Hu, Z. Chen, B. Yang, Y. Liu, X. Gao, Q. Wang, Z. Chen, Y. Liu, X. Wang, Y. Liu, In-situ bonding enables robust fluorinated poly(imide-siloxane) based triboelectric nanogenerator for firefighter motion and location monitoring, Nano Energy 138 (2025) 110856. https://doi.org/10.1016/j.nanoen.2025.110856.

[18] B. Zhou, X. Bu, J. Li, C. Gao, X. Saitaer, J. Guo, Electro–centrifugal spinning of core–sheath composite yarns with micro/nano structures for self–powered sensing, Composites Communications 53 (2025) 102141. https://doi.org/10.1016/j.coco.2024.102141.

[19] S. Qiu, X. Teng, Y. Zhang, X. Wang, K. Chen, J. Zhao, Q. Huang, Wearing comfortable and high electrical output TENGs woven with PTFE core–shell nanofiber yarns, Chemical Engineering Journal 505 (2025) 159501. https://doi.org/10.1016/j.cej.2025.159501.
